# Blimp-1 and c-Maf regulate immune gene networks to protect against distinct pathways of pathobiont-induced colitis

**DOI:** 10.1038/s41590-024-01814-z

**Published:** 2024-04-12

**Authors:** Marisol Alvarez-Martinez, Luke S. Cox, Claire F. Pearson, William J. Branchett, Probir Chakravarty, Xuemei Wu, Hubert Slawinski, Alaa Al-Dibouni, Vasileios A. Samelis, Leona Gabryšová, Simon L. Priestnall, Alejandro Suárez-Bonnet, Anna Mikolajczak, James Briscoe, Fiona Powrie, Anne O’Garra

**Affiliations:** 1 Immunoregulation and Infection Laboratory, The Francis Crick Institute, London, UK; 2 Kennedy Institute of Rheumatology, University of Oxford, Oxford, UK; 3 Computational Biology Laboratory, The Francis Crick Institute, London, UK; 4 Advanced Sequencing Facility, The Francis Crick Institute, London, UK; 5 Department of Pathobiology and Population Sciences, Royal Veterinary College, London, UK; 6 Experimental Histopathology, The Francis Crick Institute, London, UK; 7 Developmental Dynamics Laboratory, The Francis Crick Institute, London, UK; 8 National Heart and Lung Institute, Imperial College London, London, UK

**Keywords:** T cells, Monocytes and macrophages, Inflammatory bowel disease

## Abstract

Intestinal immune responses to microbes are controlled by the cytokine IL-10 to avoid immune pathology. Here, we use single-cell RNA sequencing of colon lamina propria leukocytes (LPLs) along with RNA-seq and ATAC-seq of purified CD4^+^ T cells to show that the transcription factors Blimp-1 (encoded by *Prdm1*) and c-Maf co-dominantly regulate *Il10* while negatively regulating proinflammatory cytokines in effector T cells. Double-deficient *Prdm1*^fl/fl^*Maf*^fl/fl^*Cd4*^Cre^ mice infected with *Helicobacter hepaticus* developed severe colitis with an increase in T_H_1/NK/ILC1 effector genes in LPLs, while *Prdm1*^fl/fl^*Cd4*^Cre^ and *Maf*^fl/fl^*Cd4*^Cre^ mice exhibited moderate pathology and a less-marked type 1 effector response. LPLs from infected *Maf*^fl/fl^*Cd4*^Cre^ mice had increased type 17 responses with increased *Il17a* and *Il22* expression and an increase in granulocytes and myeloid cell numbers, resulting in increased T cell–myeloid–neutrophil interactions. Genes over-expressed in human inflammatory bowel disease showed differential expression in LPLs from infected mice in the absence of *Prdm1* or *Maf*, revealing potential mechanisms of human disease.

## Abstract (web summary)

In this Resource, the authors integrate multiomics data to show the effect of the transcription factors Blimp-1 and c-Maf on IL-10 and type 1 and 17 responses, which together protect against pathobiont-induced colitis.

## Main

The immune response has evolved to protect the host against infection; however, mechanisms such as the cytokine IL-10 are in place to regulate immune responses to pathogens and pathobionts to prevent untoward inflammation and host damage^1–5^. Mice deficient in IL-10 (*Il10*^*−*^^*/*^^*−*^) can develop colitis^6^, although less evidently in specific-pathogen-free-reared *Il10*^*−/*^^*−*^ mice or germ-free mice^7^, triggered by pathobionts such as *Helicobacter hepaticus* (*H. hepaticus*)^8^. T cell-derived IL-10 dominantly controls intestinal responses with T cell-specific IL-10 mutant mice developing colitis to a similar level as *Il10*^*−/*^^*−*^ mice^9^. Rare loss-of-function mutations in *Il10, Il10ra* or *Il10rb* genes result in inflammatory bowel disease (IBD) in childhood, although it is unclear whether infection by pathobionts contributes to these pathologies^10^. Genome-wide association studies have identified more than 230 loci linked to human IBD, including those associated with proinflammatory cytokines and transcription factors upstream of immune effector molecules^11^, such as Blimp-1, encoded by *Prdm1* (ref. ^12^).

Both common and cell-specific transcriptional mechanisms regulate *Il10* and proinflammatory gene expression in T cells to ensure a controlled immune response to pathogens or other triggers^1,3–5,13–15^. Given that transcription factors have multiple gene targets, those that positively regulate *Il10* may simultaneously repress proinflammatory cytokine expression in T cells. c-Maf induces *Il10* expression directly in multiple T cell subsets, both in vitro and in vivo^1^, while also acting as a negative regulator of *Il2* (ref. ^13^) and other proinflammatory cytokines^16^ and also exhibiting context-specific effects^13^. Deletion of *Maf* in T cells or regulatory T cells (T_reg_) was reported to not result in spontaneous inflammation^13,17^, whereas in other studies, mice with T cell or T_reg_-specific deletion of *Maf* showed signs of intestinal inflammation^18,19^. The transcription factors c-Maf and Blimp-1 are dominant shared coregulators of *Il10* gene expression in multiple T cell subsets^14,15^. Although T cell-specific deletion of *Prdm1* has been reported to result in spontaneous colitis^20–22^ associated with increased frequencies of T helper 17 (T_H_17) cells^23^, other studies reported no intestinal inflammation in these mice^14,24^. Moreover, although Blimp-1 functions as a molecular switch to prevent inflammatory activity in Foxp3^+^RORγt^+^ T_reg_^25^, deletion of *Prdm1* in T_reg_ did not result in severe intestinal inflammation^26^. Spontaneous colitis has been reported in mice with T cell-specific deletion of the combination of both *Prdm1* and *Maf*, and this pathology was associated with a unique cluster of T_reg_ cells and the abrogation of *Il10* expression^14^.

We report here that mice with T cell-specific deletion of *Prdm1, Maf* or both transcription factors do not develop colitis at the steady state. Upon infection with *H. hepaticus*, the absence of *Prdm1* or *Maf* in T cells resulted in mild to moderate pathology, while the absence of both transcription factors resulted in severe pathology. We interrogated the immune response in the colon LPLs underpinning the pathology in the different T cell-specific transcription-factor-deficient *H.* *hepaticus*-infected mice using bulk tissue RNA sequencing (RNA-seq) and single-cell RNA sequencing (scRNA-seq), complemented by RNA-seq and assay for transposase-accessible chromatin sequencing (ATAC-seq) analysis of purified CD4^+^ T cells, and we validated key findings using flow cytometry and immunofluorescence staining of colon tissue. Double-deficient *Prdm1*^fl/fl^*Maf*^fl/fl^*Cd4*^Cre^ *H. hepaticus*-infected mice showed a major increase in genes associated with T_H_1 and natural killer/innate lymphoid cell 1 (NK/ILC1) effector function including interferon-γ (IFNγ) and granulocyte–macrophage colony-stimulating factor (GM-CSF), but this was lower in *Prdm1*^fl/fl^*Cd4*^Cre^ and *Maf*^fl/fl^*Cd4*^Cre^ mice. By contrast, LPLs from *H. hepaticus*-infected *Maf*^fl/fl^*Cd4*^Cre^ mice showed an increased type 17 response with increased expression of *Il17a* and *Il22* and a pronounced signature of innate immunity and neutrophils. Genes identified as over-expressed in human IBD colon biopsies from transcriptomic datasets were differentially perturbed in the LPLs of *H. hepaticus*-infected mice with T cell-specific deficiencies in either *Prdm1, Maf* or both transcription factors, potentially reflecting different pathobiological mechanisms relevant to human IBD.

## Results

### T cell Blimp-1 and c-Maf control colitis via lymphoid and myeloid cells

**Fig. 1:**
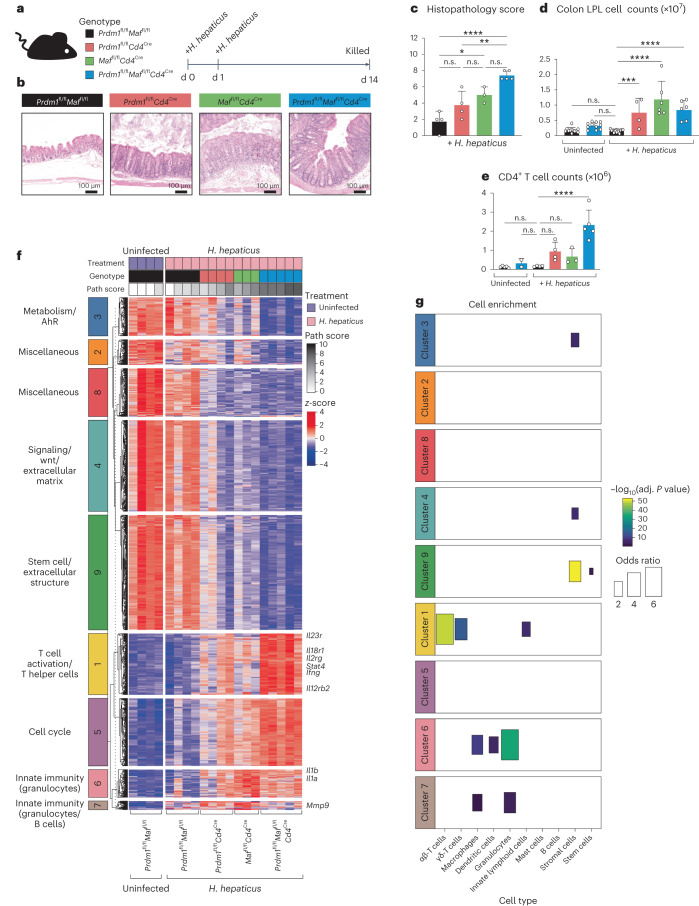
T cell-specific Blimp-1 and c-Maf control intestinal responses. **a**, Schematic of experimental method used to infect mice with *H. hepaticus* by oral gavage. **b–e**, Representative H&E colon sections from each genotype following infection with *H. hepaticus* for 14 days (**b**) with the corresponding colon histopathology scores (detailed in Methods) (**c**), colon LPL cell counts for each *H.* *hepaticus*-infected group compared to uninfected controls (**d**) and total CD4^+^ T cell counts for each *H. hepaticus*-infected group compared to uninfected controls (**e**). Each dot within the bar plots represents an individual mouse analyzed. Graph shows means, error bars, s.d. Analyzed by one-way ANOVA followed by Dunnett post-hoc test (*P ≤ 0.05, **P ≤ 0.01, ***P ≤ 0.001, ****P ≤ 0.0001). Scale bar, 100 μm. Bulk tissue RNA-seq was performed on total colon LPLs isolated from uninfected *Prdm1*^fl/fl^*Maf*^fl/fl^ control and *H. hepaticus*-infected *Prdm1*^fl/fl^*Maf*^fl/fl^ mice as well as mice with *Cd4*^Cre^-mediated deletion of either *Prdm1*, *Maf* or both *Prdm1* and *Maf*. **f**, Heatmap of expression values (represented as *z*-scores) of DEGss identified in *H. hepaticus*-infected mice compared to uninfected *Prdm1*^fl/fl^*Maf*^fl/fl^ controls (fold change ≥1.5 and Benjamini–Hochberg (BH)-adjusted *P* < 0.05), partitioned into nine clusters using *k*-means clustering. Pathology scores associated with each mouse are shown at the top of the heatmap. **g**, Enrichment of cell-type signatures (taken from a previous publication^51^) was assessed for each of the clusters in **f** using a Fisher’s exact test. Only statistically significant enriched signatures (BH-adjusted *P* < 0.05) were plotted for visualization. Data from *n* = 3–5 mice.

**Extended Data Fig. 1:**
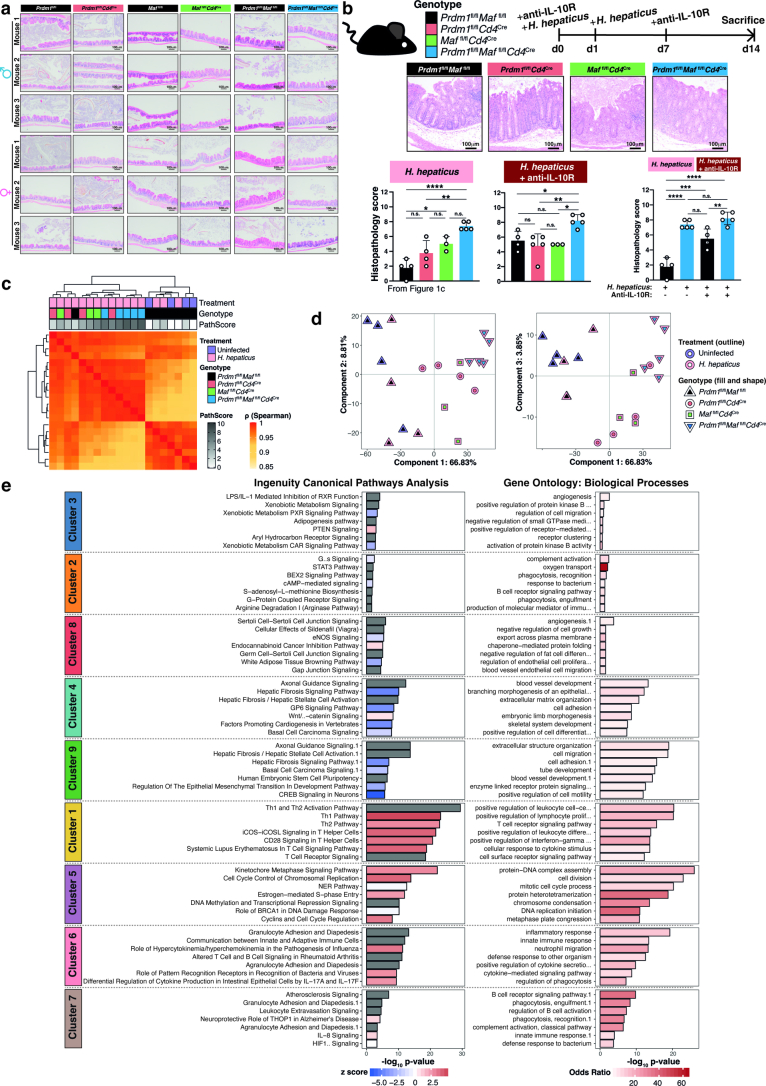
Changes in gene expression regulated by Blimp-1 and c-Maf. **a)** Representative H&E colon sections from steady state mice aged to 24-30 weeks, scale bar = 100μm. Data representative of multiple female and male mice: *Prdm1*^fl/fl^ (n = 8), *Prdm1*^fl/fl^*Cd4*^Cre^ (n = 19), *Maf*^fl/fl^ (n = 10), *Maf*^fl/fl^*Cd4*^Cre^ (n = 22), *Prdm1*^fl/fl^*Maf*^fl/fl^ (n = 36), *Prdm1*^fl/fl^*Maf*^fl/fl^*Cd4*^*C*re^ (n = 38) **b)** Schematic of experimental method used to infect mice with *H. hepaticus* by oral gavage and treatment with anti-IL-10R blocking antibody. Representative H&E colon sections (scale bar = 100μm) from each genotype following infection with *H. hepaticus* and treatment with anti-IL-10R antibody and harvested on Day 14 with the corresponding colon histopathology scores (bottom panels). Each dot within the barplots represents an individual mouse analyzed. Graph shows mean ±s.d., analyzed by one-way ANOVA followed by Tukey’s post-hoc test (*=p value ≤ 0.05, **=p value ≤ 0.01, ***=p value ≤ 0.001, ****=p value ≤ 0.0001). **c)** Unsupervised hierarchical clustering of a pair-wise Spearman correlation and **d)** PCA plots showing on PC1 the separation of floxed control mice (uninfected and infected) versus *H.* hepaticus-infected mice with *Cd4*^Cre^-mediated deletion of *Prdm1*, *Maf*, or *Prdm1* and *Maf*. **e)** Enriched IPA canonical pathways (left) and gene ontology biological processes (right) were obtained for the clusters of differentially expressed genes arising upon infection and *Cd4*^Cre^-mediated deletion of either *Prdm1*, *Maf*, or both *Prdm1* and *Maf* (related to Fig. 1f). Data from n = 3^−^5 mice.

*Prdm1*^fl/fl^*Cd4*^Cre^, *Maf*^fl/fl^*Cd4*^Cre^ and *Prdm1*^fl/fl^*Maf*^fl/fl^*Cd4*^Cre^ mice did not develop colitis in the steady state (Extended Data Fig. 1a). Upon infection with *H. hepaticus*, these T cell-specific transcription-factor-deficient mice developed colitis with varying degrees of pathology, with an overall trend of double-deficient *Prdm1*^fl/fl^*Maf*^fl/fl^*Cd4*^Cre^ mice developing the most severe disease, and the *Prdm1*^fl/fl^*Cd4*^Cre^ and *Maf*^fl/fl^*Cd4*^Cre^ mice each developing mild to moderate colitis, respectively compared to *Prdm1*^fl/fl^*Maf*^fl/fl^ control mice (hereafter referred to as control mice), which showed no inflammation or colitis (Fig. 1a–c and Methods). Total LPLs increased in all three knockouts compared to controls (Fig. 1d), and CD4^+^ T cell numbers were increased in the infected single *Prdm1*^fl/fl^*Cd4*^Cre^ and *Maf*^fl/fl^*Cd4*^Cre^ and most significantly increased in the double-deficient *Prdm1*^fl/fl^*Maf*^fl/fl^*Cd4*^Cre^ mice compared to controls (Fig. 1e).

Given that Blimp-1 and c-Maf induce *Il10* gene expression^14,15^ while negatively regulating a large network of proinflammatory cytokines^15^, we determined whether the effects of T cell-specific deletion of *Prdm1, Maf* or the combination of *Prdm1 and Maf* resulted in increased pathology and inflammation in *H. hepaticus*-infected mice owing to abrogation of IL-10 signaling. To address this question, the T cell-specific transcription-factor-deficient and control mice were infected with *H. hepaticus* in the presence of an anti-IL-10R blocking antibody (mAb) or isotype-matched mAb control (Extended Data Fig. 1b, right-hand side). Blockade of IL-10R signaling resulted in moderate to severe pathology in the colons of wild-type control mice and resulted in increased pathology in the single *Prdm1*^fl/fl^*Cd4*^Cre^ and *Maf*^fl/fl^*Cd4*^Cre^ *H. hepaticus*-infected mice. However, the most severe pathology was still observed in the infected double-deficient *Prdm1*^fl/fl^*Maf*^fl/fl^*Cd4*^Cre^ mice in the presence or absence of anti-IL-10R mAb, with no significant increase in pathology observed in mice administered anti-IL-10R mAb compared to those given isotype-control mAb (Extended Data Fig. 1b, right-hand side). These findings suggest that the high level of intestinal pathology observed in the *Prdm1*^fl/fl^*Maf*^fl/fl^*Cd4*^Cre^ mice results from the effects of both transcription factors on other immune factors in addition to their co-dominant role in *Il10* gene regulation.

To dissect the mechanisms underlying the pathology observed in the different T cell-specific transcription-factor-deficient mice, we performed RNA-seq analysis on LPLs from *H. hepaticus*-infected and uninfected mice (Extended Data Fig. 1c,d, Fig. 1f and Supplementary Table 1). *Prdm1*^fl/fl^*Cd4*^Cre^ and *Maf*^fl/fl^*Cd4*^Cre^ showed a major increase in differentially expressed genes (DEGs) against uninfected control mice, while double-deficient *Prdm1*^fl/fl^*Maf*^fl/fl^*Cd4*^Cre^ mice showed much higher numbers of DEGs compared to infected controls, which showed minimal DEGs (Supplementary Table 2). These formed nine clusters of similarly regulated DEGs, which were annotated using pathway analysis tools (Fig. 1f, Supplementary Table 2 and Extended Data Fig. 1e) with the associated pathology scores shown at the top of the heatmap (Fig. 1f). DEGs in cluster 3 (Metabolism/AhR), cluster 4 (signaling/Wnt/extracellular matrix), cluster 9 (stem cell/extracellular structure) and clusters 2 and 8 (miscellaneous) were all decreased in the LPLs from the *H. hepaticus*-infected transcription-factor-deficient mice compared to control mice and largely represented non-immune genes (Fig. 1f). Conversely, DEGs in cluster 1 (T cell activation/T helper cells) and cluster 5 (cell cycle) were partially increased in LPLs from both *Prdm1*^fl/fl^*Cd4*^Cre^ and *Maf*^fl/fl^*Cd4*^Cre^ *H. hepaticus*-infected mice, and further increased in the double-deficient *Prdm1*^fl/fl^*Maf*^fl/fl^*Cd4*^Cre^ mice (Fig. 1f). DEGs in cluster 6 (innate immunity/granulocytes) and cluster 7 (innate immunity/granulocytes/B cells) were most markedly increased in *H. hepaticus*-infected *Maf*^fl/fl^*Cd4*^Cre^ mice and in the double-deficient *Prdm1*^fl/fl^*Maf*^fl/fl^*Cd4*^Cre^ mice, albeit to a lesser extent, while barely increased in the infected *Prdm1*^fl/fl^*Cd4*^Cre^ (Fig. 1f and Supplementary Table 2). Enrichment of cell type-specific gene signatures, derived using ImmGen Ultra Low Input data (GSE109125), validated the pathway annotation of the clusters representing immune pathways (Fig. 1g and Extended Data Fig. 1e). Cluster 1 (T cell activation/T helper cells) was enriched in αβ-T cells, γδ-T cells and ILCs, and cluster 6 (innate immunity) and cluster 7 (granulocyte-associated genes) showed enrichment of macrophages, dendritic cells and granulocytes, and macrophages and granulocyte-associated genes, respectively (Fig. 1g).

**Fig. 2:**
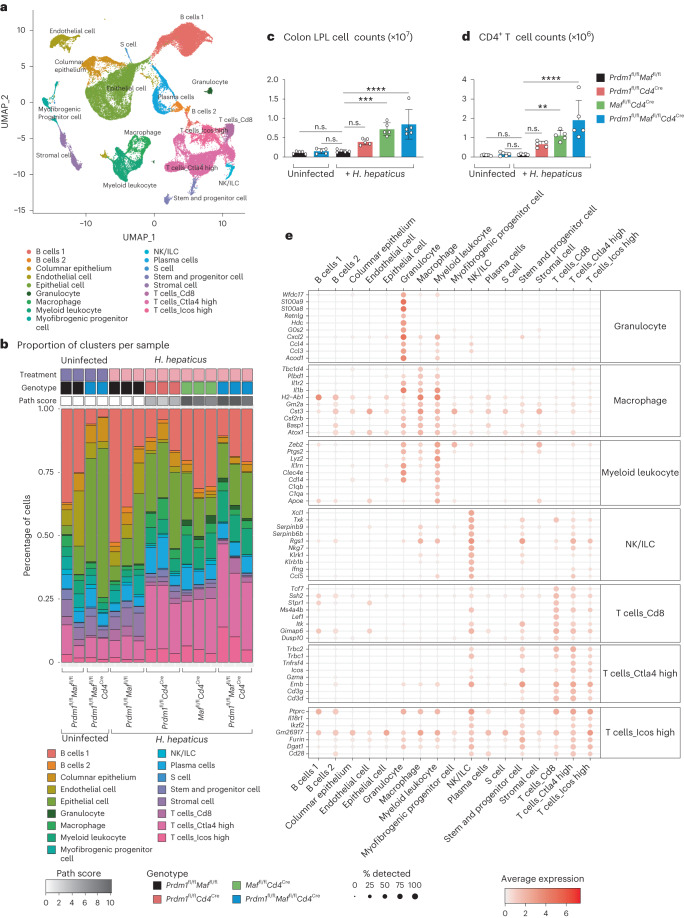
scRNA-seq reveals in-depth colonic gene regulation by *Prdm1* and *Maf*. scRNA-seq was performed on total colon LPL isolated from uninfected *Prdm1*^fl/fl^*Maf*^fl/fl^ and *Prdm1*^fl/fl^*Maf*^fl/fl^*Cd4*^*C*re^ control mice, and *H. hepaticus*-infected *Prdm1*^fl/fl^*Maf*^fl/fl^ mice as well as mice with *Cd4*^Cre^-mediated deletion of either *Prdm1*, *Maf* or both *Prdm1* and *Maf*. **a**, Uniform manifold approximation and projection (UMAP) visualization of the integrated scRNA-seq from all conditions, colored by the identified/assigned cell cluster. **b**, Bar plots representing the proportion of cells in each of the cell clusters per biological replicate within each experimental condition and corresponding histopathological scores (detailed in Methods). **c**, Colon LPL cell counts for each *H. hepaticus*-infected group compared to uninfected controls. **d**, Total CD4^+^ T cell counts for each group in the experiment for each *H. hepaticus*-infected group compared to uninfected controls. Each dot within the bar plots represents an individual mouse analyzed. Graph shows means; error bars, s.d. Analyzed by one-way ANOVA followed by Dunnett post-hoc test (*P ≤ 0.05; **P ≤ 0.01; ***P ≤ 0.001; ****P ≤ 0.0001). Data from *n* = 5 mice. **e**, Dot plot of the top ten differentially expressed marker genes of relevant cell clusters from **a**, colored by the average gene expression across all cell clusters. The dot size represents the percentage of cells per cell cluster expressing the gene in question.

**Extended Data Fig. 2:**
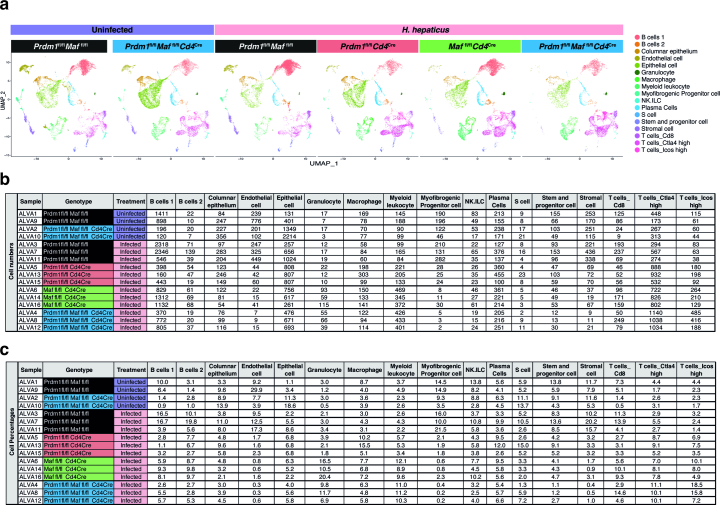
Details of colon scRNA-seq changes by *Prdm1* and *Maf*, or both. **a-c)** scRNA-seq was performed on total colon LPL isolated from uninfected *Prdm1*^fl/fl^*Maf*^fl/fl^ and *Prdm1*^fl/fl^*Maf*^fl/fl^*Cd4*^Cre^ control mice, and *H. hepaticus* infected *Prdm1*^fl/fl^*Maf*^fl/fl^ mice and mice with *Cd4*^Cre^-mediated deletion of either *Prdm1*, *Maf*, or both *Prdm1* and *Maf*. **a)** UMAP visualization of the integrated scRNA-seq dataset, plotted per condition and colored by assigned cell cluster. **b)** Total cell number and **c)** proportion of cells (%) successfully sequenced and passing quality control (Methods section) in each of the cell clusters identified. Data from n = 2-3 mice.

To interrogate the gene expression changes further and identify the cellular sources of immune-associated genes, we performed scRNA-seq on LPLs isolated from colons from an independent experiment with *H. hepaticus*-infected and uninfected mice (Fig. 2 and Extended Data Fig. 2). Data from the LPLs of all groups were first integrated for analysis into a single uniform manifold approximation and projection plot, revealing 17 distinct cell clusters (Fig. 2a) for annotation, using the single-cell Mouse Cell Atlas as in the Methods, the Immgen database (GSE109125) and manual curation (Fig. 2a and Supplementary Table 3). scRNA-seq data from LPLs from *H. hepaticus*-infected *Prdm1*^fl/fl^*Cd4*^Cre^, *Maf*^fl/fl^*Cd4*^Cre^ and double-deficient *Prdm1*^fl/fl^*Maf*^fl/fl^*Cd4*^Cre^ mice showed distinct profiles compared to uninfected (fl/fl control mice and double-deficient *Prdm1*^fl/fl^*Maf*^fl/fl^*Cd4*^Cre^ mice) and infected control fl/fl mice (Fig. 2b and Extended Data Fig. 2). A similar proportion of immune cell types was identified by scRNA-seq in the LPLs from the uninfected control mice, uninfected double-deficient *Prdm1*^fl/fl^*Maf*^fl/fl^*Cd4*^Cre+^ mice and *H. hepaticus*-infected control mice, with no intestinal pathology (Fig. 2b and Extended Data Fig. 2). LPL preparations from uninfected double-deficient *Prdm1*^fl/fl^*Maf*^fl/fl^*Cd4*^Cre+^ mice, however, showed some unexplainable increases in the proportion of epithelial cells, although these mice showed no intestinal pathology (Fig. 2b; see Methods). Moreover, no increase in LPLs (Fig. 2c) or CD4^+^ T cells (Fig. 2d) assessed by flow cytometry was observed at the steady state in these mice.

**Extended Data Fig. 3:**
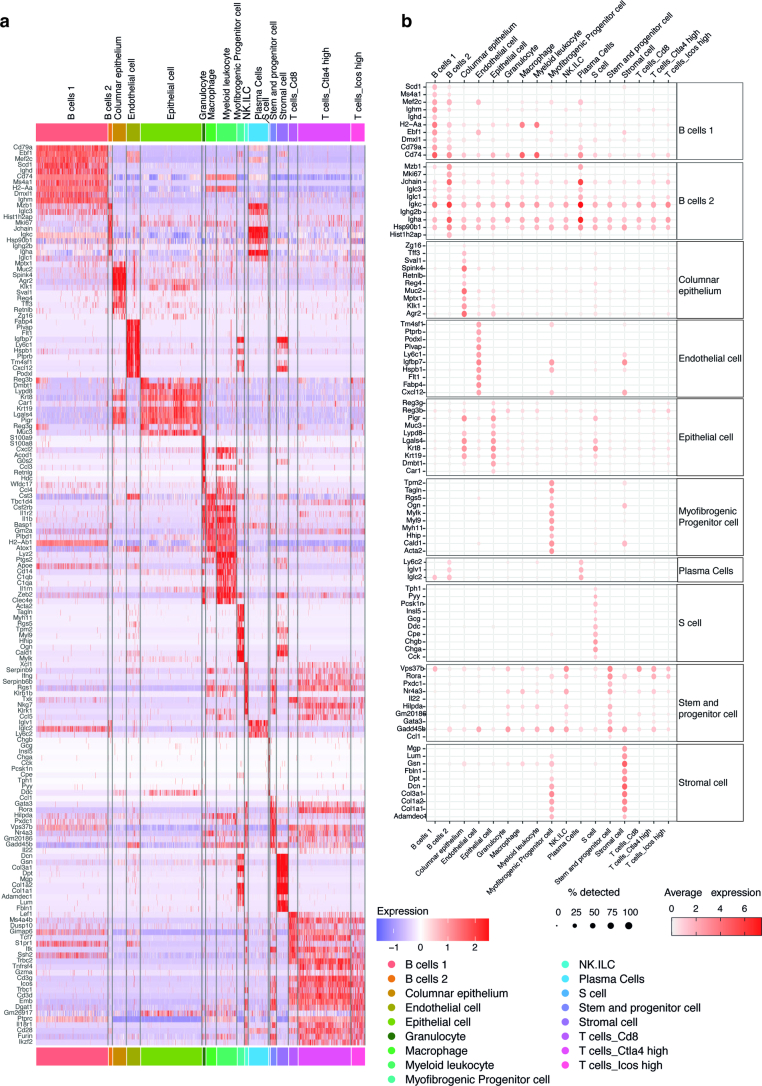
Top genes in colon scRNAseq clusters in infected mice. **a)** Heatmap and **b)** dotplot (of cell clusters not in Fig. 2e) of the top 10 differentially expressed marker genes of the identified cell clusters in our colon LPL scRNA-seq. Colored by the average gene expression across all cell clusters. In the dotplots, the dot size represents the percentage of cells per cell cluster expressing the gene in question.

Increased intestinal pathology (Fig. 2b) and LPL and CD4^+^ T cell numbers were again observed by flow cytometry (Fig. 2c,d) in *H. hepaticus*-infected *Prdm1*^fl/fl^*Cd4*^Cre^, *Maf*^fl/fl^*Cd4*^Cre^ and double-deficient *Prdm1*^fl/fl^*Maf*^fl/fl^*Cd4*^Cre^ compared to control fl/fl mice. The scRNA-seq data revealed a similar increase in the proportion of T cells but additionally revealed greater granularity, with an increase in a subset of *Ctla4* high T cells in the LPL from *Prdm1*^fl/fl^*Cd4*^Cre^ and *Maf*^fl/fl^*Cd4*^Cre^ mice and to a greater extent in infected double-deficient *Prdm1*^fl/fl^*Maf*^fl/fl^*Cd4*^Cre^ mice (Fig. 2b, Extended Data Fig. 2 and Supplementary Table 3). A smaller increase of *Icos* high T cells but not Cd8 T cells was observed (Fig. 2b and Extended Data Fig. 2). The NK/ILC cell cluster expressed a distinct discrete set of NK cell-specific genes, including *Nkg7, Klrb1b* and the highest level of *Ifng*, confirming their identity as NK/ILC1 cells (Fig. 2e and Extended Data Fig. 3a). The Ctla4 high and Icos high T cell subsets, although clustering separately, broadly shared the expression of the top ten marker genes (Fig. 2e and Extended Data Fig. 3a). scRNA-seq data recapitulated an increase in granulocytes in *H. hepaticus*-infected *Maf*^fl/fl^*Cd4*^Cre^ and double-deficient *Prdm1*^fl/fl^*Maf*^fl/fl^*Cd4*^Cre^ only, but not in infected *Prdm1*^fl/fl^*Cd4*^Cre^ or control mice (Fig. 2b), with increased expression of genes associated with neutrophils, including *Acod1, S100a8* and *S100a9* (Fig. 2e, Extended Data Fig. 3a and Supplementary Table 3). Populations annotated as macrophages and myeloid leukocytes were increased in the LPLs from *H. hepaticus*-infected *Prdm1*^fl/fl^*Cd4*^Cre^, *Maf*^fl/fl^*Cd4*^Cre^ and double-deficient *Prdm1*^fl/fl^*Maf*^fl/fl^*Cd4*^Cre^ mice (Fig. 2b and Extended Data Fig. 2), with increased expression of genes associated with myeloid or innate immune responses such as *Lyz2 (LysM), Csf2rb, Il1r2, Il1b* and *Cd14* (Fig. 2e, Extended Data Fig. 3a and Supplementary Table 3). These scRNA-seq data thus identified the cellular sources of key gene expression signatures.

### *Prdm1* and *Maf* induce *Il10* while also disrupting effector T cell gene expression

**Fig. 3:**
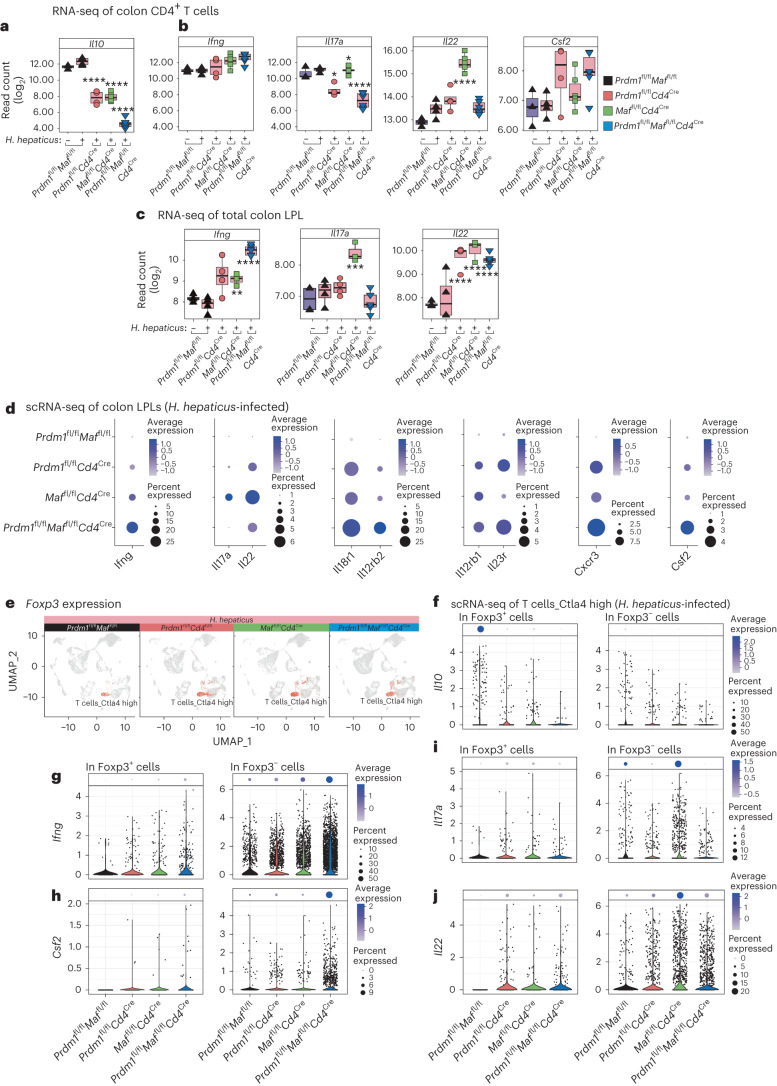
*Prdm1* and *Maf* induce *Il10 and* control T effector cytokines. **a**,**b**, RNA-seq gene expression of *Il10* (**a**) and *Ifng, Il17a, Il22* and *Csf2* (**b**) in sorted CD4^+^ T cells from colon LPLs isolated from uninfected *Prdm1*^fl/fl^*Maf*^fl/fl^ mice and *H. hepaticus*-infected *Prdm1*^fl/fl^*Maf*^fl/fl^ mice as well as mice with *Cd4*^Cre^-mediated deletion of either *Prdm1*, *Maf* or both *Prdm1* and *Maf*. **c**, Gene expression of *Ifng, Il17a* and *Il22* in bulk tissue total colon LPLs isolated from uninfected and *H. hepaticus*-infected mice. In **a**–**c**, DEGs in each condition against uninfected *Prdm1*^fl/fl^*Maf*^fl/fl^ mice were marked for statistical significance as follows, *BH-adjusted *P* ≤ 0.05; **BH-adjusted *P* ≤ 0.01; ***BH-adjusted *P* ≤ 0.001; ****BH-adjusted *P* ≤ 0.0001. **d**, Dot plot of scRNA-seq gene expression of selected genes *Ifng, Il17a, Il22, Il18r1, Il12rb2, Il12rb1, Il23r, Cxcr3* and *Csf2* in colon LPLs from *H.* *hepaticus*-infected *Prdm1*^fl/fl^*Maf*^fl/fl^ or mice with *Cd4*^Cre^-mediated deletion of either *Prdm1*, *Maf* or both *Prdm1* and *Maf*. The dot size represents the percentage of cells per cell cluster expressing the gene in question and the expression level indicated by the color scale. **e**, Expression of *Foxp3* as assessed by scRNA-seq within the UMAP visualization of annotated scRNA-seq datasets within each of *H.* *hepaticus*-infected genotypes. **f**–**j**, Expression at the single-cell level of *Il10* (**f**), *Ifng* (**g**), *Csf2* (**h**), *Il17a* (**i**) and *Il22* (**j**) in the 'Ctla4 high' cells expressing *Foxp3* (Foxp3^+^) or not (Foxp3^−^). The distribution of expression in cells within each condition is shown by the violin plots; the dot plot (top panels) displays the proportion of cells expressing the gene in question, with the expression level indicated by the color scale.

**Extended Data Fig. 4:**
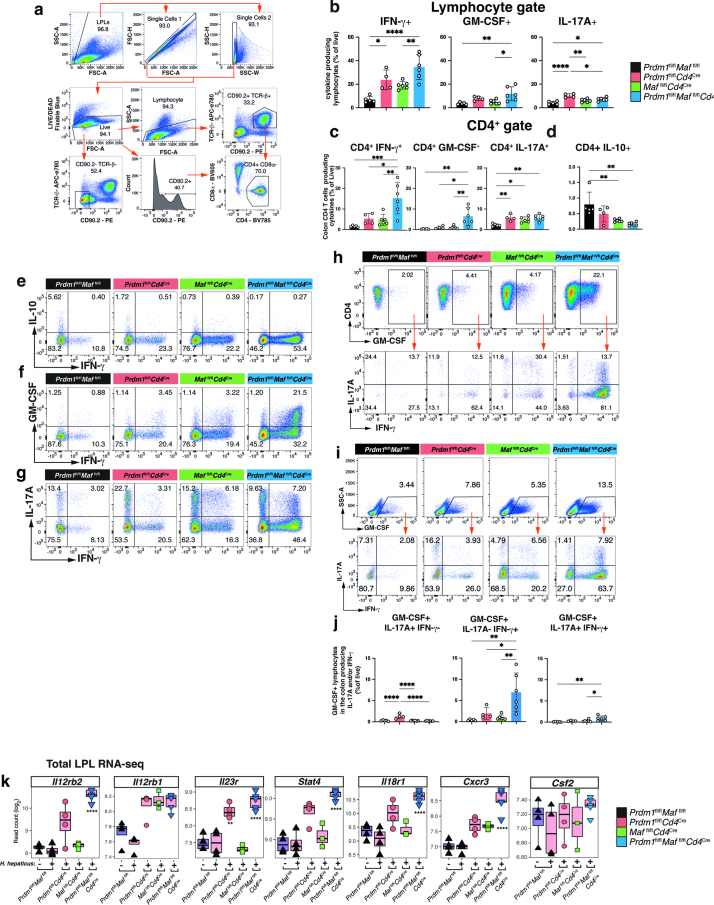
Flow cytometry and RNAseq analysis of colon in infected mice. **a)** Gating strategy for flow cytometry analysis of colon LPLs. **b–d)** Percentages from live cells of **b)** total lymphocytes and **c-d)** total CD4 + T cells (Live CD90.2 + TCR-β + CD4 + CD8-)) producing IFN-γ, GM-CSF, IL-17A and IL-10. Flow plots of CD4 + T-cells for IFN-γ production versus **e)** IL-10, **f)** GM-CSF and **g)** IL-17A. **h)** Gating of total CD4 + GM-CSF+ lymphocytes with corresponding flow plots of IFN-γ versus IL-17A. **i)** Gating of total GM-CSF+ lymphocytes with corresponding flow plots of IFN-γ versus IL-17A with bar plots of **j)** percentages (from live population) of lymphocytes producing GM-CSF alongside IL-17A, IFN-γ, or both. All flow analyses were performed on from *H. hepaticus* infected mice. **k)** Gene expression of *Il12rb2, Il12rb1, Il23r, Stat4, Il18r1, Cxcr3* and *Csf2* in bulk tissue total colon LPL isolated from uninfected and *H. hepaticus* infected mice. Differentially expressed genes in each condition against uninfected *Prdm1*^fl/fl^*Maf*^fl/fl^ mice were marked as follows: *=BH adjusted p value ≤ 0.05, **= BH adjusted p value ≤ 0.01, ***= BH adjusted p value ≤ 0.001, ****= BH adjusted p value ≤ 0.0001.

Expression of *Il10* mRNA in purified CD4^+^ T cells from LPLs was diminished in *H. hepaticus*-infected *Prdm1*^fl/fl^*Cd4*^Cre^ and *Maf*^fl/fl^*Cd4*^Cre^ mice, and to the greatest extent in the double-deficient *Prdm1*^fl/fl^*Maf*^fl/fl^*Cd4*^Cre^ mice (Fig. 3a and Supplementary Tables 4 and 5). This was mirrored by similar decreases in IL-10 protein production (Extended Data Fig. 4a,d,e). Conversely, CD4^+^ T cells showed increased *Ifng* expression in infected *Prdm1*^fl/fl^*Cd4*^Cre^ and *Maf*^fl/fl^*Cd4*^Cre^ mice (Fig. 3b), with the greatest increase observed in the double-deficient *Prdm1*^fl/fl^*Maf*^fl/fl^*Cd4*^Cre^, mirrored by increased IFNγ protein production (Extended Data Fig. 4a–c,e). Increased *Ifng* expression was more pronounced in total LPLs by RNA-seq (Fig. 3c) and scRNA-seq (Fig. 3d) than in CD4^+^ T cells, suggesting that increased numbers of CD4^+^ T cells, γδ-T cells or NK/ILC1 are contributing to the global increased levels of *Ifng* expression in the LPLs of the *H.* *hepaticus*-infected T cell-specific transcription-factor-deficient mice. The level of *Il17a* and *Il22* expression in purified CD4^+^ T cells from the LPLs was highest in the infected *Maf*^fl/fl^*Cd4*^Cre^ mice and lower in those from infected *Prdm1*^fl/fl^*Maf*^fl/fl^*Cd4*^Cre^ and *Prdm1*^fl/fl^*Cd4*^Cre^ mice (Fig. 3b), with similar findings in LPLs by RNA-seq (Fig. 3c) and scRNA-seq (Fig. 3d). Levels of *Il17a were* similar in CD4^+^ T cells in the infected *Maf*^fl/fl^*Cd4*^Cre^ mice to those in uninfected and infected control mice (Fig. 3b). However, scRNA-seq showed exclusive elevation of *Il17a* at the level of expression and percentage of *Il17a* expressing cells, suggesting that increased numbers of CD4^+^ T cells, γδ-T cells or ILC3 contribute to the global increase in *Il17a* expression in LPLs from *H. hepaticus*-infected *Maf*^fl/fl^*Cd4*^Cre^ mice (Fig. 3c,d). *Csf2* RNA expression was highest in CD4^+^ T cells (Fig. 3b) and in scRNA-seq of LPLs from infected double-deficient *Prdm1*^fl/fl^*Maf*^fl/fl^*Cd4*^Cre^ mice (Fig. 3d), as were T_H_1-associated effector molecules *Ifng, Il18r1, Il12rb2, Il12rb1* and *Cxcr3*, and additionally *Il23r*. Many of these genes were also elevated in the *Prdm1*^fl/fl^*Cd4*^Cre^ but not in the *Maf*^fl/fl^*Cd4*^Cre^ mice (Fig. 3b,d and Extended Data Fig. 4k).

**Extended Data Fig. 5:**
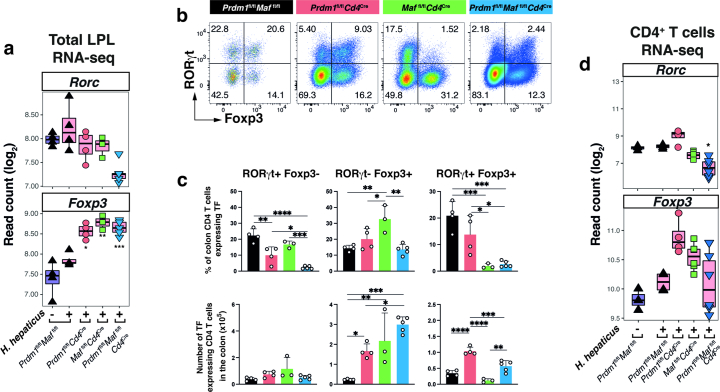
c-Maf regulates colon RORγt+Foxp3+ cells during infection. **a)** Boxplots of log2(normalized read counts) of *Rorc* and *Foxp3* in bulk tissue LPL RNA-seq. **b)** Flow cytometry analysis of RORγt and Foxp3 transcription factor expression with **c)** percentages (top) and numbers (bottom) of CD4 + T cells producing of RORγt and/or Foxp3. **d)** Boxplots of log2(normalized read counts) of *Rorc* and *Foxp3* in sorted CD4 + T cells from colon LPL isolated from uninfected and *H. hepaticus* infected mice. Differentially expressed genes in each condition against uninfected *Prdm1*^fl/fl^*Maf*^fl/fl^ mice were marked as follows: *=BH adjusted p value ≤ 0.05, **= BH adjusted p value ≤ 0.01, ***= BH adjusted p value ≤ 0.001, ****= BH adjusted p value ≤ 0.0001.

The scRNA-seq data was further interrogated to identify the source of cells expressing *Il10* and proinflammatory cytokines in the LPLs from the *H. hepaticus*-infected T cell-specific transcription-factor-deficient mice. Foxp3^+^ T_reg_ cells contained within the Ctla4 high cluster of T cells (Fig. 3j) were identified as the main *Il10*-expressing T cells (Fig. 3e). Expression and percentages of *Il10*-producing cells were diminished in Foxp3^+^ T_reg_ cells from both the *Prdm1*^fl/fl^*Cd4*^Cre^, *Maf*^fl/fl^*Cd4*^Cre^ and to the greatest extent in the double-deficient *Prdm1*^fl/fl^*Maf*^fl/^^fl^*Cd4*^Cre^ infected mice compared to infected control mice, and reduced similarly in the very low numbers of Foxp3^−^CD4^+^ *Il10*-expressing T cells (Fig. 3f). Foxp3^+^ T_reg_ cells showed a graded increase in numbers by flow cytometry in the LPLs from *H. hepaticus*-infected *Prdm1*^fl/fl^*Cd4*^Cre^, *Maf*^fl/fl^*Cd4*^Cre^ and double-deficient *Prdm1*^fl/fl^*Maf*^fl/fl^*Cd4*^Cre^ mice, respectively (Extended Data Fig. 5a–c). By contrast, Foxp3^+^RORγt^+^ T cells were almost completely abolished in the LPLs from infected *Maf*^fl/fl^*Cd4*^Cre^ mice (Extended Data Fig. 5b,c) as previously reported^27^, whereas they were increased in LPLs from infected *Prdm1*^fl/fl^*Cd4*^Cre^ and to a lesser extent double-deficient *Prdm1*^fl/fl^*Maf*^fl/fl^*Cd4*^Cre^ mice compared to control mice (Extended Data Fig. 5b,c). Despite this, infected double-deficient *Prdm1*^fl/fl^*Maf*^fl/^^fl^*Cd4*^Cre^ mice exhibited the maximum pathology (Fig. 1b,c and Fig. 2b) and expressed the lowest levels of *Il10* in CD4^+^ T cells (Fig. 3a,f).

In contrast to the dominant expression of *Il10* in Foxp3^+^ T_reg_ cells, *Ifng* and *Csf2* were most highly expressed in the Foxp3^−^ Ctla4 high T cell cluster, while being scarcely detectable in Foxp3^+^ T_reg_ cells (Fig. 3g,h) and increased in *Prdm1*^fl/fl^*Cd4*^Cre^ and *Maf*^fl/fl^*Cd4*^Cre^ and mostly highly in double-deficient *Prdm1*^fl/fl^*Maf*^fl/fl^*Cd4*^Cre^-infected mice, respectively. Conversely, *Il17a* and *Il22* expression by scRNA-seq, while also expressed largely in Foxp3^−^CD4^+^ T cells, was highest in LPLs from infected *Maf*^fl/fl^*Cd4*^Cre^ mice (Fig. 3i,j), in keeping with RNA-seq data from flow cytometry-purified CD4^+^ T cells (Fig. 3b). Diminished expression of *Il17a* and *Il22* in LPLs from infected *Prdm1*^fl/fl^*Maf*^fl/^^fl^*Cd4*^Cre^ mice suggest repression of the *Il17a* and/or *Il22* response by Blimp-1 regulated factors (Fig. 3i,j). Collectively, these data suggest that the intestinal pathology resulting from T cell-specific deficiency in *Maf* differs qualitatively from that of *Prdm1* and even *Prdm1–Maf* double deficiency owing to a type 17-mediated effector cytokine response to *H. hepaticus* infection rather than the increased type 1 mediated response controlled by both transcription factors.

**Fig. 4:**
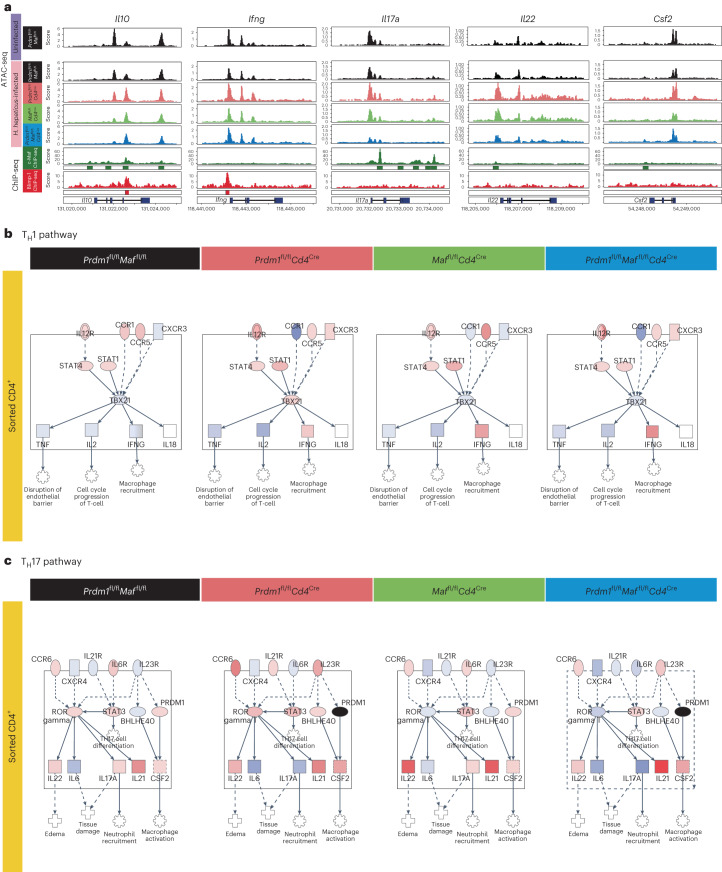
Multi-omic integration identifies binding sites for Blimp-1 and c-Maf. **a**, ATAC-seq was performed on sorted CD4^+^ T cells from colon LPLs isolated from uninfected *Prdm1*^fl/fl^*Maf*^fl/fl^ control and *H. hepaticus*-infected *Prdm1*^fl/fl^*Maf*^fl/fl^ mice as well as mice with *Cd4*^Cre^-mediated deletion of either *Prdm1*, *Maf* or both *Prdm1* and *Maf*. Genome browser tracks of ATAC-seq data from each condition together with publicly available c-Maf (green) and Blimp-1 (red) ChIP-seq datasets in the *Il10*, *Ifng*, *Il17a, Il22* and *Csf2* loci. Statistically significant ChIP-seq peaks (*q* < 0.05) are represented by a green or red bar underneath the normalized read coverage tracks for c-Maf and Blimp-1, respectively. **b**,**c**, IPA was used to overlay sorted CD4^+^ T cells RNA-seq onto the T_H_1 (**b**) and T_H_17 (**c**) pathways. Gene expression fold changes in each *H.* *hepaticus*-infected condition relative to the uninfected *Prdm1*^fl/fl^*Maf*^fl/fl^ control were overlayed onto the T_H_1 and T_H_17 pathways. A fixed scale of −5 (blue) to 3.5 (red) was kept between all conditions, and *Prdm1* was colored black in the *Prdm1*-deficient T cells.

**Extended Data Fig. 6:**
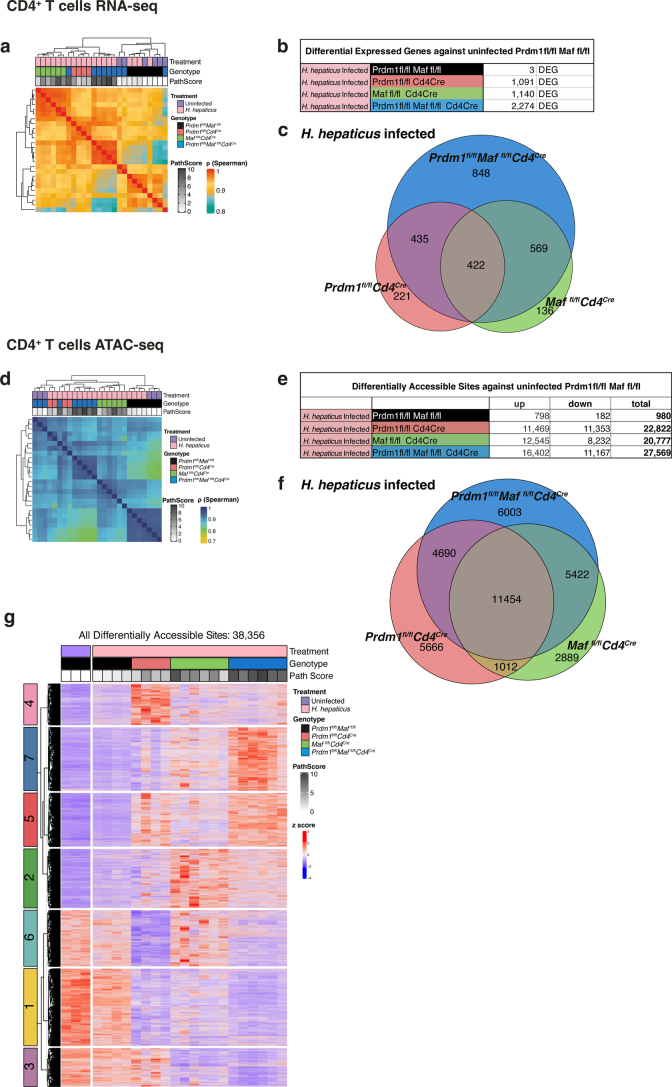
Changes in T-cell gene expression and chromatin accessibility. **a)** Unsupervised hierarchical clustering of a pair-wise Spearman correlation of RNA-seq on sorted CD4 + T cells from colon LPL isolated from uninfected *Prdm1*^fl/fl^*Maf*^fl/fl^ and *Prdm1*^fl/fl^*Maf*^fl/fl^*Cd4*^*C*re^, and *H. hepaticus* infected *Prdm1*^fl/fl^*Maf*^fl/fl^ mice and mice with *Cd4*^Cre^-mediated deletion of either *Prdm1*, *Maf*, or both *Prdm1* and *Maf*. **b)** Number of differentially expressed genes identified in *H. hepaticus* infected mice compared to uninfected *Prdm1*^fl/fl^*Maf*^fl/fl^ controls (fold change >=1.5 and BH adjusted p value < 0.05). **c)** Euler diagram of distinct and overlapping differentially expressed genes between transcription factor-deficient T cells from *H. hepaticus* infected mice. **d)** Unsupervised hierarchical clustering of a pair-wise Spearman correlation of ATAC-seq on sorted CD4 + T cells from colon LPL isolated from uninfected *Prdm1*^fl/fl^*Maf*^fl/fl^ and *Prdm1*^fl/fl^*Maf*^fl/fl^*Cd4*^*C*re^, and *H. hepaticus* infected *Prdm1*^fl/fl^*Maf*^fl/fl^ mice and mice with *Cd4*^Cre^-mediated deletion of either *Prdm1*, *Maf*, or both *Prdm1* and *Maf*. **e)** Number of differentially accessible sites identified in *H. hepaticus* infected mice compared to uninfected *Prdm1*^fl/fl^*Maf*^fl/fl^ controls (fold change >=1.5 and BH adjusted p value < 0.05). **f)** Euler diagram of distinct and overlapping differentially accessible sites between transcription factor-deficient T cells from *H. hepaticus* infected mice. **g)** Heatmap of accessibility values (represented as z-scores) of accessible sites identified in CD4 + T cells from *H. hepaticus* infected mice compared to uninfected *Prdm1*^fl/fl^*Maf*^fl/fl^ controls (fold change >=1.5 and BH adjusted p value < 0.05), partitioned into 7 clusters using *k*-means clustering. Pathology scores associated to each mouse are shown at the top of the heatmap.

Global analysis of RNA-seq and ATAC-seq data revealed large unique and overlapping changes in gene expression and differentially accessible sites in CD4^+^ T cells from both *H. hepaticus*-infected *Prdm1*^fl/fl^*Cd4*^Cre^ and *Maf*^fl/fl^*Cd4*^Cre^ mice compared to control mice, with much higher changes in CD4^+^ T cells from infected double-deficient *Prdm1*^fl/fl^*Maf*^fl/fl^*Cd4*^Cre^ mice (Extended Data Fig. 6a–g and Supplementary Tables 6 and 7). A combination of ATAC-seq and RNA-seq data from purified CD4^+^ T cells from colon LPLs of the *H. hepaticus*-infected T cell-specific transcription-factor-deficient mice was integrated with published chromatin immunoprecipitation with sequencing (ChIP-seq) data and revealed common and distinct binding sites for Blimp-1 and c-Maf in the *Il10* locus as previously reported^14^. However, distinct binding sites for Blimp-1 in the *Ifng* locus were observed, while c-Maf had several binding sites within the *Il17* locus and weak binding sites in the *Il22* and *Csf2* gene loci (Fig. 4a). Our data are supportive of cooperative and independent roles of these transcription factors in the positive regulation of *Il10* and additionally in negative regulation of proinflammatory cytokine genes. Pathway analysis applied to the RNA-seq data from CD4^+^ T cells revealed an increase in IL-12R and downstream Stat1/Stat4 signaling for *Ifng* induction in the CD4^+^ T cells from the *H. hepaticus-*infected *Prdm1*^fl/fl^*Cd4*^Cre^, *Maf*^fl/fl^*Cd4*^Cre^ and double-deficient *Prdm1*^fl/fl^*Maf*^fl/fl^*Cd4*^Cre^ mice, respectively, with the largest increase in IFNγ observed in the double-deficient mice compared to infected control mice (Fig. 4b). Pathway analysis showed the highest increase of IL17A and IL22 within the T_H_17 pathway in the *Maf*^fl/fl^*Cd4*^Cre^ CD4^+^ T cells, with a marked decrease in infected double-deficient *Prdm1*^fl/fl^*Maf*^fl/fl^*Cd4*^Cre^ mice, suggesting that factors regulated by Blimp-1 may repress expression of the *Il17a* gene (Fig. 4c). Expression of *Csf2* observed within the T_H_17 pathway was again increased in CD4^+^ T cells from LPLs of all the *H. hepaticus*-infected *Prdm1*^fl/fl^*Cd4*^Cre^, *Maf*^fl/fl^*Cd4*^Cre^ although most marked in *Prdm1*^fl/fl^*Maf*^fl/fl^*Cd4*^Cre^ mice compared to control infected mice (Fig. 4c).

### T cell-derived Blimp-1 and c-Maf control innate immunity

**Fig. 5:**
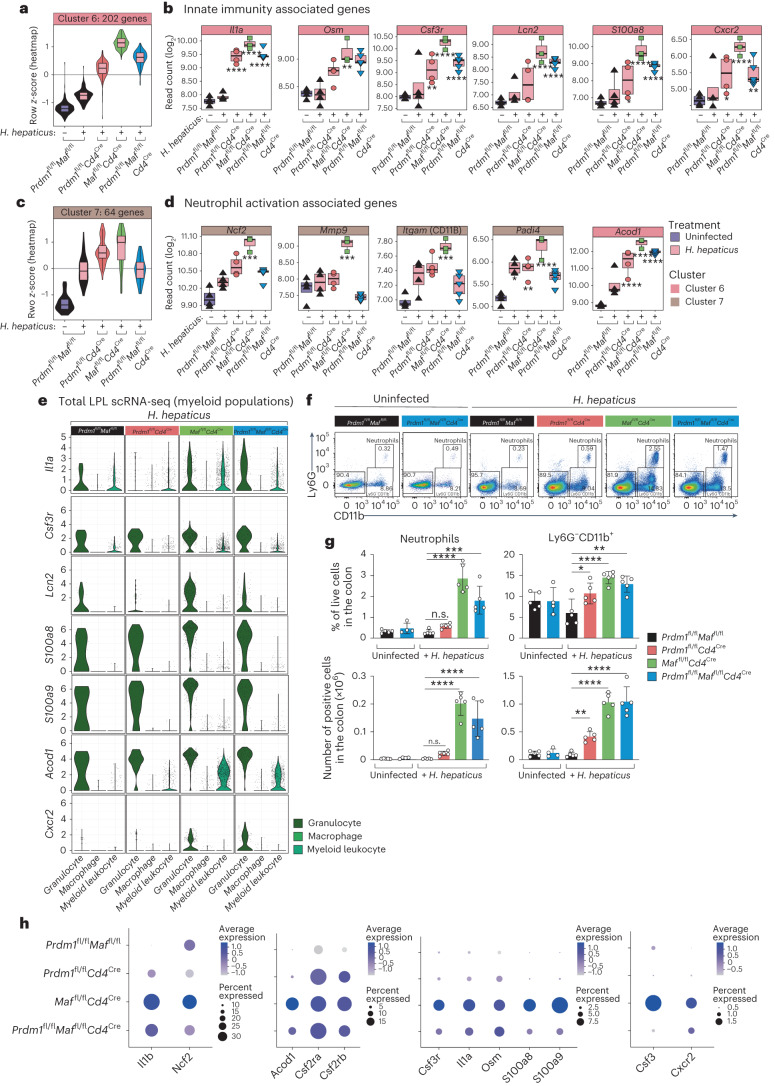
Increased myeloid gene expression in the absence of *Maf* in T cells. **a**–**d**, Violin plots summarizing the expression values (quantified as *z*-scores) and boxplots of example genes of innate immunity-associated genes found in cluster 6 (**a**,**b**) and neutrophil activation associated genes found in cluster 7 (**c**,**d**) of the bulk tissue LPL RNA-seq data analysis from Fig. 1f. DEGs in each condition against uninfected *Prdm1*^fl/fl^*Maf*^fl/fl^ mice were marked as follows (all *P* values BH-adjusted): *P ≤ 0.05; **P ≤ 0.01; ***P ≤ 0.001; ****P ≤ 0.0001. **e**, Expression of selected innate immunity and granulocyte-associated genes queried in the granulocyte, macrophage and myeloid leukocyte cell clusters across all *H.* *hepaticus*-infected conditions within the colon LPL scRNA-seq dataset. **f**, Representative flow plots and gating strategy used for the flow cytometry analysis of neutrophils (Live CD90.2^−^TCR-β^−^CD19^−^ CD11^+^Ly6G^+^) across uninfected *Prdm1*^fl/fl^*Maf*^fl/fl^ and *Prdm1*^fl/fl^*Maf*^fl/fl^*Cd4*^Cre^, and *H.* *hepaticus*-infected *Prdm1*^fl/fl^*Maf*^fl/fl^ mice as well as mice with *Cd4*^Cre^-mediated deletion of either *Prdm1*, *Maf* or both *Prdm1* and *Maf*. **g**, Bar plots of percentage from live (top panels) and absolute cell counts (bottom panels) of neutrophils and Ly6G^–^CD11b^+^ cells from lamina propria of the colon. Each dot within the bar plots represents an individual mouse analyzed. Graph shows means, error bars, s.d. Analyzed by one-way ANOVA followed by Dunnett post-hoc test (*P ≤ 0.05; **P ≤ 0.01; ***P ≤ 0.001; ****P ≤ 0.0001). Data from *n* = 4–5 mice. **h**, Dot plot of scRNA-seq gene expression of selected genes *Il1b, Ncf2, Acod1, Csf2ra, Csf2rb, Csf3r, Il1a, Osm, S100a8, S100a9, Csf3 and Cxcr2*, in the colon LPLs from *H.* *hepaticus*-infected control *Prdm1*^fl/fl^*Maf*^fl/fl^ mice and mice with *Cd4*^Cre^-mediated deletion of either *Prdm1*, *Maf* or both *Prdm1* and *Maf*. The dot size represents the percentage of cells per cell cluster expressing the gene in question, and the expression level is indicated by the color scale.

The average increased expression of ‘innate immunity and myeloid-associated’ genes in the bulk tissue RNA-seq data, cluster 6 (Fig. 1f,g) from the LPLs from *H. hepaticus*-infected *Maf*^fl/fl^*Cd4*^Cre^ and, to a lesser extent, *Prdm1*^fl/fl^*Cd4*^Cre^ and double-deficient *Prdm1*^fl/fl^*Maf*^fl/fl^*Cd4*^Cre^ mice compared to controls was confirmed quantitatively (Fig. 5a); for example, for *Il1a* and *Osm*, encoding Oncostatin M (Fig. 5b), which was previously associated with colitis^11,28,29^. Granulocyte-associated genes in this cluster, including *Csf3r, Lcn2, S100a8* and *Cxcr2*, were most highly expressed in the LPLs from infected *Maf*^fl/fl^*Cd4*^Cre^ mice and to a much lesser extent in the double-deficient *Prdm1*^fl/fl^*Maf*^fl/fl^*Cd4*^Cre^ and *Prdm1*^fl/fl^*Cd4*^Cre^ mice (Fig. 5b). Similarly, while the average gene expression in the bulk tissue RNA-seq data granulocyte cluster 7 (Fig. 1f,g) was increased in the LPLs of control mice upon infection with *H. hepaticus*, a further increase was seen in the *Maf*^fl/fl^*Cd4*^Cre^ and to a lesser extent in the *Prdm1*^fl/fl^*Cd4*^Cre^ infected mice, but not in the double-deficient *Prdm1*^fl/fl^*Maf*^fl/fl^*Cd4*^Cre^ infected mice, which showed levels similar to those of the *H. hepaticus*-infected control mice (Fig. 5c). Genes associated with granulocyte/neutrophil activation, including *Ncf2*, *Itgam, Mmp9* and *Padi4*, showed the highest expression in the LPLs from infected *Maf*^fl/fl^*Cd4*^Cre^ mice but not in the *Prdm1*^fl/fl^*Cd4*^Cre^ or double-deficient *Prdm1*^fl/fl^*Maf*^fl/fl^*Cd4*^Cre^ infected mice, which showed similar or reduced levels to those from infected control mice, the latter suggesting potential counter-regulatory mechanisms provided by Blimp-1 signaling in T cells (Fig. 5d). Additionally, *Acod1*, a gene encoding enzyme aconitate decarboxylase 1 (Irg1), which produces the metabolite itaconate in myeloid cells^30^, was found to be most highly expressed in LPLs from infected *Maf*^fl/fl^*Cd4*^Cre^ mice and double-deficient *Prdm1*^fl/fl^*Maf*^fl/fl^*Cd4*^Cre^ infected mice (Fig. 5d). scRNA-seq analysis showed that the granulocyte/neutrophil and myeloid leukocyte clusters were the source of *Il1a* and *Acod1* in the LPLs from infected *Maf*^fl/fl^*Cd4*^Cre^ and double-deficient *Prdm1*^fl/fl^*Maf*^fl/fl^*Cd4*^Cre^ mice (Fig. 5e). The expression of genes associated with granulocytes/neutrophils, including *Lcn2, S100a8, S100a9* and *Cxcr2*, was exclusively detected in the ‘granulocyte/neutrophil’ cluster by scRNA-seq (Fig. 5e). Although basal expression of some of these genes was observed in the low number of granulocytes detected by scRNA-seq in the infected control mice, increased expression of these neutrophil-associated genes (Fig. 5e) and granulocyte percentage and numbers (Extended Data Fig. 2b,c) were observed in the LPLs from the *Maf*^fl/fl^*Cd4*^Cre^ and the double-deficient *Prdm1*^fl/fl^*Maf*^fl/fl^*Cd4*^Cre^ infected mice. In keeping with the scRNA-seq data, increased percentage and numbers of Ly6G^+^CD11b^+^ neutrophil cells were observed by flow cytometry analysis in the LPLs from the *Maf*^fl/fl^*Cd4*^Cre^ but to a lesser extent in double-deficient *Prdm1*^fl/fl^*Maf*^fl/fl^*Cd4*^Cre^ *H. hepaticus*-infected mice compared to uninfected or infected control mice and infected *Prdm1*^fl/fl^*Cd4*^Cre^ mice, in which these populations were barely detectable (Fig. 5f,g). This was reinforced by analysis of combined gene expression and percentage in the scRNA-seq data for granulocyte-specific genes, such as *Ncf2, Csf3r, S100a8* and *S100a9*, as well as innate cell genes *Il1a, Il1b* and *Acod1*, which were maximal in the LPLs from the infected *Maf*^fl/fl^*Cd4*^Cre^ mice (Fig. 5h). Ly6G^−^CD11b^+^ myeloid cells, while present in uninfected and infected control mice at low numbers, showed a significant increase by flow cytometry in the LPLs from the *H. hepaticus*-infected *Prdm1*^fl/f^*Cd4*^Cre^ mice, and to a greater extent in both *Maf*^fl/fl^*Cd4*^Cre^ and double-deficient *Prdm1*^fl/fl^*Maf*^fl/fl^*Cd4*^Cre^ mice compared to the uninfected or infected control mice (Fig. 5f,g). This increase in myeloid cells is in keeping with increased expression of *Il1a, Il1b, Acod1* and the *Csf2ra* and *Csf2rb* in the LPLs from both the infected *Maf*^fl/fl^*Cd4*^Cre^ and double-deficient *Prdm1*^fl/fl^*Maf*^fl/fl^*Cd4*^Cre^ mice (Fig. 5e,h).

### *Prdm1* and *Maf* control T cell–myeloid colonic cell interactions

**Fig. 6:**
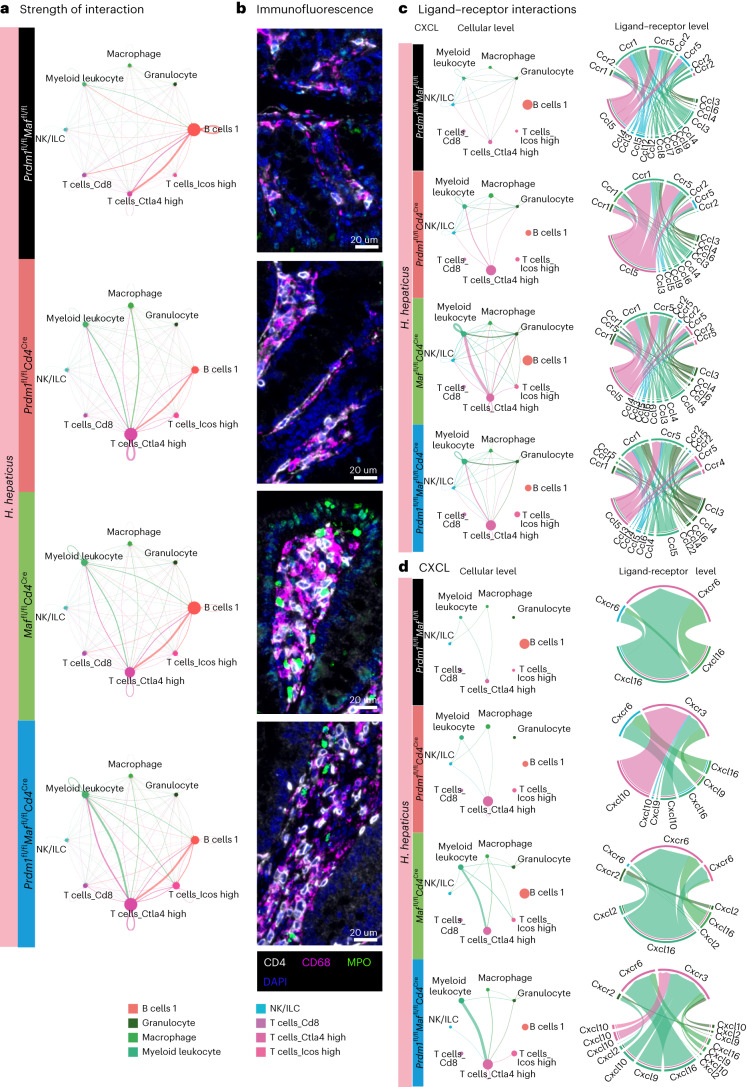
Increased interaction between T cells, macrophages and neutrophils. Cell-to-cell communication networks inferred using CellChat software from gene expression of ligands and their receptors in immune cell clusters of interest from the colonic LPL scRNA-seq dataset. **a**, Strength of interaction of cell-to-cell interactions, represented in the edge width, in *H.* *hepaticus*-infected *Prdm1*^fl/fl^*Maf*^fl/fl^ mice and mice with *Cd4*^Cre^-mediated deletion of either *Prdm1*, *Maf* or both *Prdm1* and *Maf*. **b**, Representative images (*n* = 4–5) of colon sections by immunofluorescence, staining for CD4^+^ T cells (CD4, white), mononuclear phagocytes (CD68, magenta), neutrophils (MPO, green) and nuclear staining (DAPI, blue) from *H.* *hepaticus*-infected *Prdm1*^fl/fl^*Maf*^fl/fl^ mice and mice with *Cd4*^Cre^-mediated deletion of either *Prdm1*, *Maf* or both *Prdm1* and *Maf*. Scale bar, 20 μm. **c**,**d**, Cell-to-cell communication networks underlying CCL (**c**) and CXCL (**d**) pathways across all *H.* *hepaticus*-infected *Prdm1*^fl/fl^*Maf*^fl/fl^ mice and mice with *Cd4*^Cre^-mediated deletion of either *Prdm1*, *Maf* or both *Prdm1* and *Maf*. The chord plot has receiver cells at the top (incoming signaling) and transmitter cells (outgoing signaling) at the bottom. The edges are colored based on the cell clusters expressing the outgoing signals. In **a**,**c** and **d**, node size is proportional to the number of cells in each experimental group, and the edges are colored based on the cell clusters expressing the outgoing signals.

**Extended Data Fig. 7:**
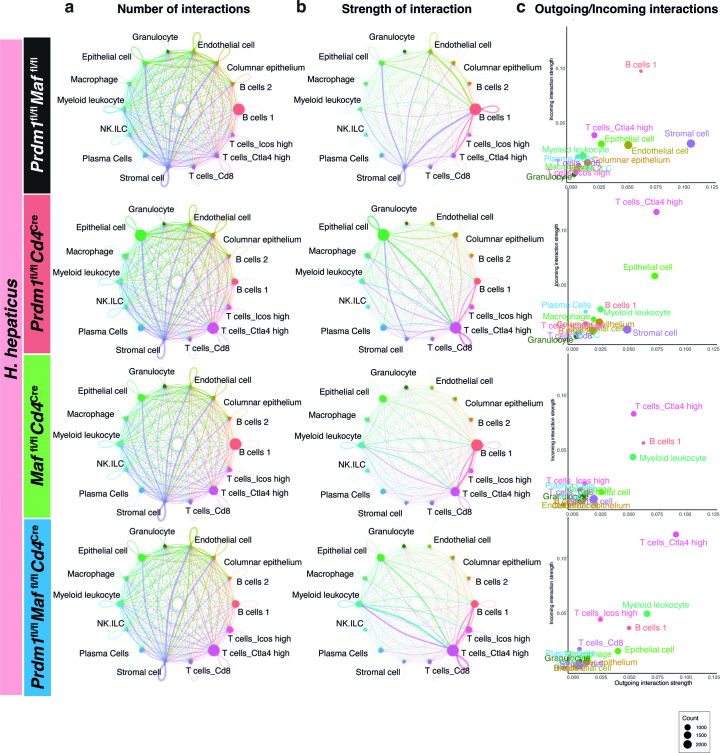
Inference of cell-to-cell communication networks in scRNA-seq. Cell-to-cell communication networks inferred using CellChat software from gene expression of ligands and their receptors in immune, stromal, epithelial and endothelial cell clusters from the colonic LPL scRNA-seq dataset. **a)** number and **b)** strength of interaction of cell-to-cell interactions, represented in the edge width, in and *H. hepaticus* infected *Prdm1*^fl/fl^*Maf*^fl/fl^ mice and mice with *Cd4*^Cre^-mediated deletion of either *Prdm1*, *Maf*, or both *Prdm1* and *Maf*. **c)** Plot of “Outgoing interaction strength“ against “Incoming interaction strength” across all *H. hepaticus* infected conditions. In **a-b)** node size is proportional to the number of cells in each experimental group, and the edges are colored based on the cell clusters expressing the outgoing signals.

**Extended Data Fig. 8:**
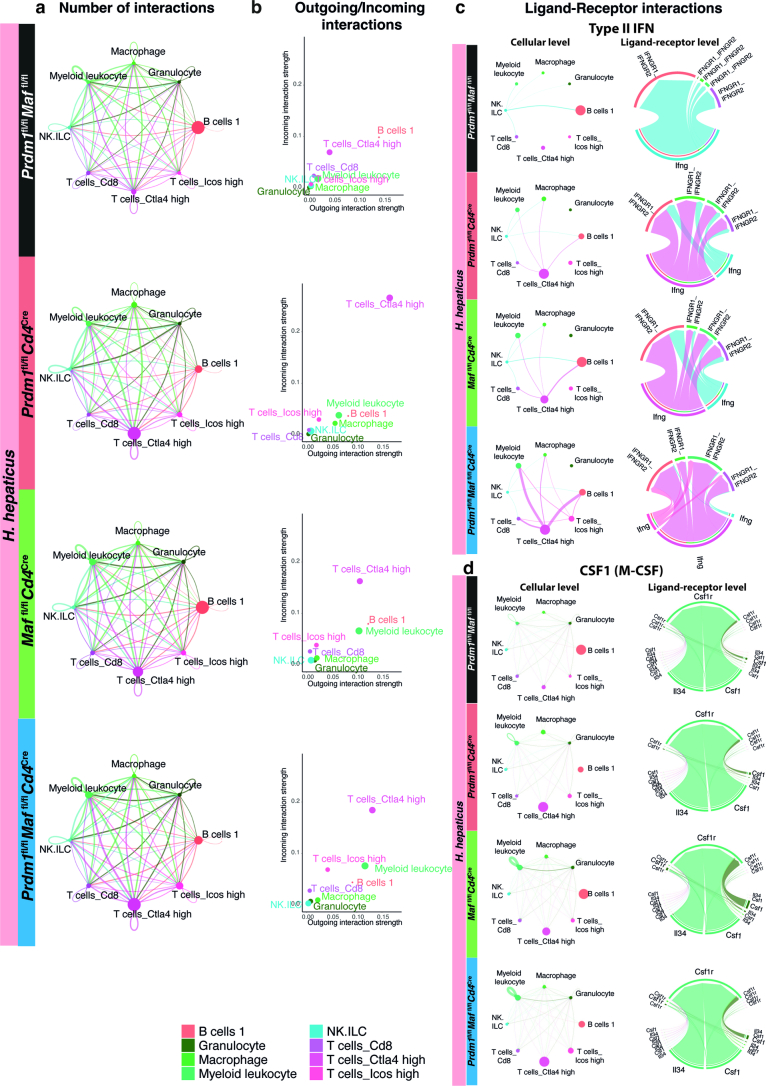
Prdm1 and Maf control T cell-IFN-γ and M-CSF pathways. Cell-to-cell communication networks inferred using CellChat software from gene expression of ligands and their receptors in immune cell clusters of interest from the colonic LPL scRNA-seq dataset. **a)** Number of interactions of cell-to-cell interactions, represented in the edge width, in and *H. hepaticus* infected *Prdm1*^fl/fl^*Maf*^fl/fl^ mice and mice with *Cd4*^Cre^-mediated deletion of either *Prdm1*, *Maf*, or both *Prdm1* and *Maf*. **b)** Plot of “Outgoing interaction strength“ against “Incoming interaction strength” across all *H. hepaticus* infected conditions. **c-d)** Cell-to-cell communication networks underlying the **c)** Type II IFN (IFN-γ) and **d)** M-CSF pathways across all *H. hepaticus* infected *Prdm1*^fl/fl^*Maf*^fl/fl^ mice and mice with *Cd4*^Cre^-mediated deletion of either *Prdm1*, *Maf*, or both *Prdm1* and *Maf*. The chord plot has receiver cells at the top (incoming signaling) and transmitter cells (outgoing signaling) the bottom. The edges are colored based on the cell clusters expressing the outgoing signals. In **a,c-d)** node size is proportional to the number of cells in each experimental group, and the edges are colored based on the cell clusters expressing the outgoing signals.

**Extended Data Fig. 9:**
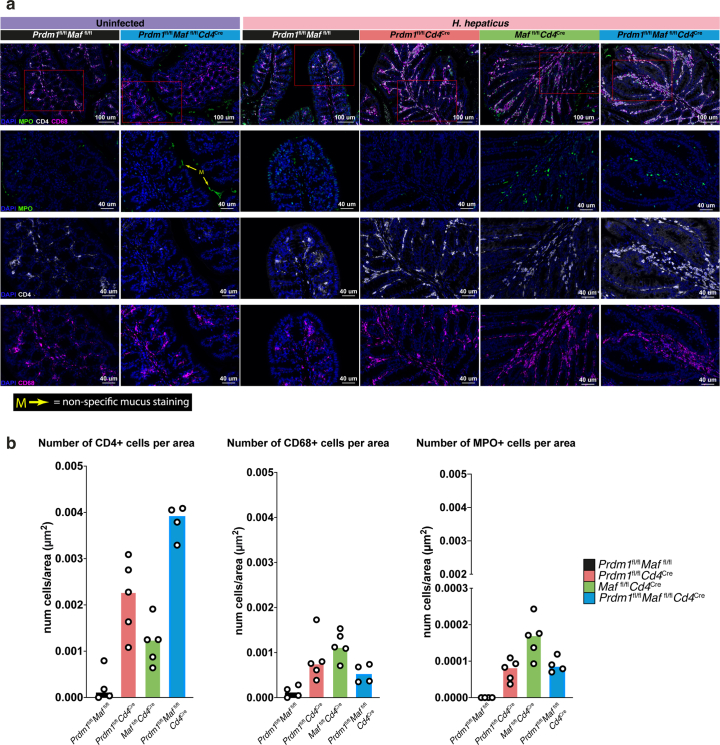
Co-localization of colon neutrophils and CD4+ cells. **a)** Representative images (n = 4-5) of colon sections by immunofluorescence, staining for CD4 + T cells (CD4, white), mononuclear phagocytes (CD68, magenta), neutrophils (MPO, green) and nuclear staining (DAPI, blue) from uninfected *Prdm1*^fl/fl^*Maf*^fl/fl^ and *Prdm1*^fl/fl^*Maf*^fl/fl^*Cd4*^*C*re^, and *H. hepaticus* infected *Prdm1*^fl/fl^*Maf*^fl/fl^ mice and mice with *Cd4*^Cre^-mediated deletion of either *Prdm1*, *Maf*, or both *Prdm1* and *Maf*. Yellow arrows with an “M” point to acellular (no DAPI staining) non-specific mucus staining. Top row shows merged channels with a scale bar = 100μm; in the second row DAPI and MPO channels are shown with a scale bar = 40μm; in the third row DAPI and CD4 channels are shown with a scale bar = 40μm; and in the bottom row DAPI and CD68 channels are shown with a scale bar = 40μm. **b)** Bar graphs showing the median number of CD4 + , CD68+ and MPO+ cells per area (μm^2^) in gut sections of each *H. hepaticus* infected *Prdm1*^fl/fl^*Maf*^fl/fl^ mice and mice with *Cd4*^Cre^-mediated deletion of either *Prdm1*, *Maf*, or both *Prdm1* and *Maf*. Data from n = 4-5 mice.

Ligand–receptor pairs identified from the scRNA-seq data using CellChat, as referenced in the Methods, were used to infer putative cell-to-cell crosstalk (Fig. 6 and Extended Data Figs. 7 and 8). Analysis of the immune cells of interest from this study revealed outgoing and incoming interactions between the T cell Ctla4 high population and B cells in the LPLs from *H. hepaticus-*infected control mice, while in the infected *Prdm1*^fl/fl^*Cd4*^Cre^, *Maf*^fl/fl^*Cd4*^Cre^ and double-deficient *Prdm1*^fl/fl^*Maf*^fl/fl^*Cd4*^Cre^ mice, myeloid leukocyte/macrophage populations delivered increased strength of signals to the T cell Ctla4 high population, which in turn signaled back to these myeloid cells (Fig. 6a). These findings were validated by immunofluorescent staining of colon sections (Fig. 6b and Extended Data Fig. 9). CD4^+^ T cells (white) and CD68^+^ mononuclear phagocytes (magenta) were found to be increased and in close proximity in *H. hepaticus*-infected *Prdm1*^fl/fl^*Cd4*^Cre^ mice, and to a greater extent in *Maf*^fl/fl^*Cd4*^Cre^ and double-deficient *Prdm1*^fl/fl^*Maf*^fl/fl^*Cd4*^Cre^-infected mice compared to control mice (Fig. 6b). Neutrophils staining positive for MPO were most abundant in *Maf*^fl/fl^*CD4*^Cre^-infected mice (Fig. 6b), in keeping with the RNA-seq and flow cytometry data, and appeared to co-localize for the most part with CD68^+^ mononuclear phagocytes and CD4^+^ T cells (Fig. 6b). The largest inferred increase in both outgoing and incoming signals observed in myeloid leukocytes was in the LPLs from infected *Maf*^fl/fl^*Cd4*^Cre^ and double-deficient *Prdm1*^fl/f^*Maf*^fl/fl^*Cd4*^Cre^ mice, which included strong outgoing CCL and CXCL signaling. The T cell Ctla4 high population was inferred to deliver a strong *Ccl5* signal to *Ccr1/Ccr5* on myeloid leukocytes (Fig. 6c,d), whereas the myeloid leukocyte population was predicted to deliver a strong *Cxcl16/Cxcl10* signal largely to *Cxcr6/Cxcr3* on the T cell Ctla4 high population, highlighting potential axes contributing to CD4 T cell accumulation in the LPLs of these mice (Fig. 6c,d). Consistent with the well-established role of *Cxcl2–Cxcr2* in neutrophil responses, inferred interactions between the myeloid leucocyte population and granulocytes through the *Cxcl2–Cxcr2* axis—and additionally, strong *Ccl* interactions within the myeloid leukocyte population and from myeloid leukocytes to granulocytes—were detected in the infected *Maf*^fl/fl^*Cd4*^Cre^ and double-deficient *Prdm1*^fl/fl^*Maf*^fl/f^*Cd4*^Cre^ mice (Fig. 6c,d), potentially contributing to the increased neutrophil and myeloid numbers in these mice. While IFNγ signaling in infected control mice was inferred to be delivered from NK cells to B cells, an outgoing signal from T cell Ctla4 high cells to myeloid cells and B cells was observed in the infected *Prdm1*^fl/fl^*Cd4*^Cre^, *Maf*^fl/fl^*Cd4*^Cre^ and double-deficient *Prdm1*^fl/fl^*Maf*^fl/fl^*Cd4*^Cre^ mice (Extended Data Fig. 8c). Inferred *Csf1* signaling between myeloid populations detected in both *Maf*^fl/fl^*Cd4*^Cre^ and double-deficient *Prdm1*^fl/f^*Maf*^fl/f^*Cd4*^Cre^ mice (Extended Data Fig. 8d) potentially contributed to the increased myeloid cells and neutrophils. Thus, dysregulated cytokine and chemokine cell–cell interaction networks downstream of disrupted transcriptional regulation in T cells by Blimp-1 and c-Maf may contribute to pathology during *H. hepaticus* infection.

### Genes expressed in human IBD are controlled by Blimp-1 and c-Maf

**Fig. 7:**
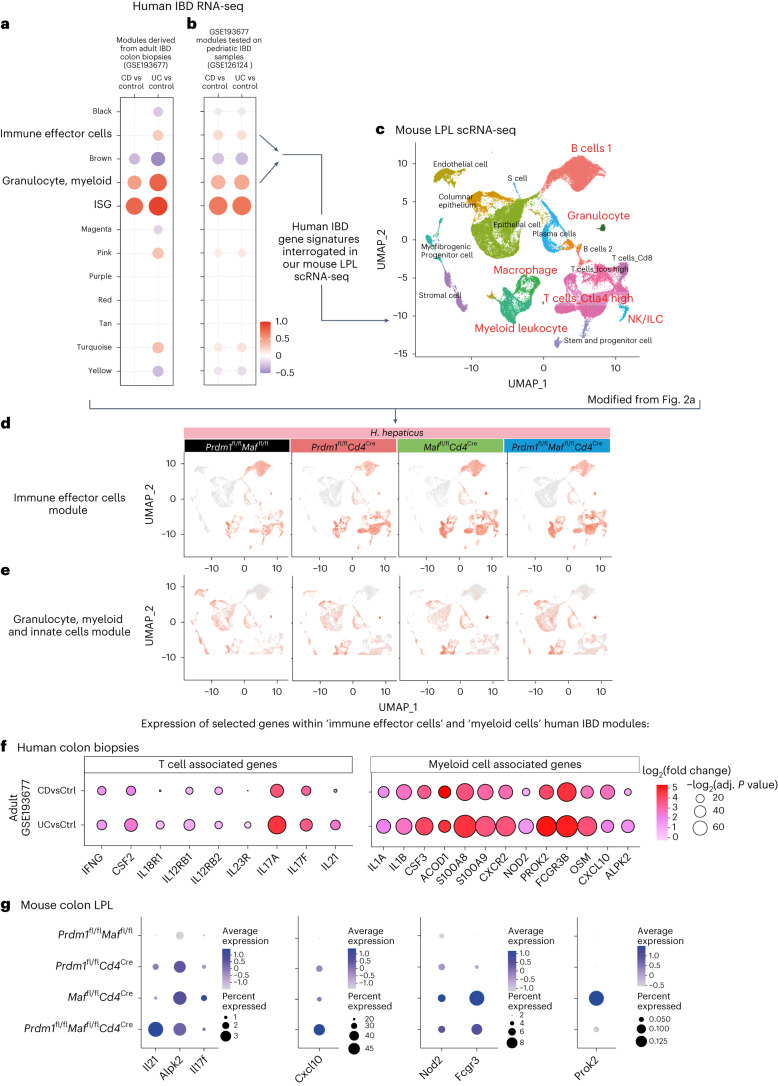
IBD patient gene signatures are regulated by *Prdm1* and *Maf.* **a**, Modules of co-expressed genes were derived from human adult IBD colonic biopsies (GSE193677) using the R package WGCNA and **b**, tested in an independent human pediatric IBD dataset (GSE126124). CD, Crohn’s disease; UC, ulcerative colitis. In the dot plots, the color and size of the dots represent the fold enrichment in disease compared to controls. Re-named modules indicate biological processes associated with the genes within a module. Fold-enrichment scores were derived using QuSAGE software; red and blue colors indicated over-abundance and under-abundance, respectively, of genes within a module (compared to control samples). Size of the dots represents the relative degree of perturbation (larger dots represent a higher degree of perturbation), and only modules with an adjusted *P* < 0.05 were considered significant and depicted in the plot. **c**, Enrichment of genes within the 'immune effector cells' and the 'granulocyte, myeloid and innate cells' modules were then tested in our mouse colon LPL scRNA-seq dataset. **d**,**e**, Scoring of the 'immune effector cells' (**d**) and 'granulocyte, myeloid and innate cells' modules (**e**) were projected into our scRNA-seq UMAP. **f**, Dot plot of over-expressed T cell-associated or myeloid cell-associated genes from human IBD colon biopsies found within the modules from **a** and **b** versus controls. The dot size represents the *P* value and the log_2_(fold change) is indicated by the color scale. **g**, Dot plot of gene expression of selected genes found to be over-expressed in human IBD as in **f**, in colon LPL scRNA-seq from *H.* *hepaticus*-infected control *Prdm1*^fl/fl^*Maf*^fl/fl^ mice and mice with *Cd4*^Cre^-mediated deletion of either *Prdm1*, *Maf* or both *Prdm1* and *Maf*. The dot size represents the percentage of cells per cell cluster expressing the gene in question and the expression level indicated by the color scale.

Transcriptomic data from colon biopsies of patients with IBD, including Crohn’s disease and ulcerative colitis, were obtained from the Gene Expression Omnibus (GEO) (GSE193677 and GSE126124). Modular analysis was performed to display clusters of up-regulated or down-regulated genes compared to controls. Modules of interest were annotated and selected to interrogate our mouse LPL scRNA-seq dataset from the *H. hepaticus*-infected mice. (Fig. 7a,b,c and Supplementary Table 8). Genes within the human IBD ‘Immune effector’ module were found to be largely expressed in the ‘B cells 1’, ‘macrophage/myeloid leukocyte’, ‘T cells Ctla4 high’ and ‘T cells Icos high’ in the mouse LPL scRNA-seq clusters from infected *Prdm1*^fl/f^*Cd4*^Cre^ mice and to a greater extent the *Maf*^fl/fl^*Cd4*^Cre^ and double-deficient *Prdm1*^fl/f^*Maf*^fl/fl^*Cd4*^Cre^ mice (Fig. 7d). Expression of genes from the human ‘granulocyte, myeloid’ module, which were more highly expressed in adult ulcerative colitis, which usually manifests as inflammation of the colon, was largely confined to the mouse scRNA-seq ‘epithelial’, ‘granulocyte’ and ‘macrophage/myeloid leukocyte’ clusters, and expressed at the highest level in the *Maf*^fl/fl^*Cd4*^Cre^ and double-deficient *Prdm1*^fl/f^*Maf*^fl/f^*Cd4*^Cre^ mice (Fig. 7e,f). T cell/ILC-related genes shown earlier to be expressed at the highest level in the LPLs from *H. hepaticus* double-deficient *Prdm1*^fl/f^*Maf*^fl/f^*Cd4*^Cre^-infected mice (Fig. 3) were contained within these human IBD modules and *IFNG, CSF2, IL18R1, IL-12RB1, IL12RB2* and *IL23R* expression was increased in Crohn’s disease and to a greater extent in ulcerative colitis (Fig. 7f). Additionally, identified within the human IBD ‘Immune effector module’, *IL21* was over-expressed in ulcerative colitis (Fig. 7f) and showed the highest abundance in the LPLs from *H. hepaticus* double-deficient *Prdm1*^fl/f^*Maf*^fl/f^*Cd4*^Cre^-infected mice (Fig. 7g), suggesting that IL-21 is associated with a type 1 response. T cell/ILC-associated genes most highly expressed in LPLs from infected *Maf*^fl/fl^*Cd4*^Cre^ mice (Fig. 3), including *IL17A* and *IL17F*, were highly expressed in human IBD, again to a higher extent in ulcerative colitis (Fig. 7f,g and Supplementary Table 8). Multiple genes associated with myeloid cells and innate immunity, which we showed earlier were most highly expressed in the LPLs from infected *Maf*^fl/fl^*Cd4*^Cre^ mice (Fig. 5h), were over-expressed in human IBD, including *IL1B, IL1A, CSF3, ACOD1, OSM, S100A8, S100A9* and *CXCR2* (Fig. 7f). However, *ALPK2*, over-expressed in human IBD and mostly in ulcerative colitis (Fig. 7f), was equally expressed in *Maf*^fl/fl^*Cd4*^Cre^ and double-deficient *Prdm1*^fl/f^*Maf*^fl/f^*Cd4*^Cre^ mice (Fig. 7g), while CXCL10 was increased in both ulcerative colitis and Crohn’s disease (Fig. 7f), and was highest in the double-deficient LPLs (Fig. 7g). Other genes identified within the human IBD ‘granulocyte, myeloid’ module, including *NOD2*, *FCGR3* and *PROK2*, which were most highly expressed in the biopsies from ulcerative colitis (Fig. 7f), were most highly abundant in the *Maf*^fl/fl^*Cd4*^Cre^ mice (Fig. 7g). Our findings indicate that genes that show increased expression in human IBD colon biopsies, including those with mutations that have been linked to an increased risk of Crohn’s disease and/or ulcerative colitis, such as *NOD2*, *IL23R*, *IL21* and *IFNG*^11^, are differentially abundant in the LPLs from *H. hepaticus*-infected mice with T cell-specific deletion of *Prdm1*, *Maf* or both transcription factors, suggesting that these mouse models may reflect different pathobiological mechanisms relevant in human IBD.

## Discussion

IL-10 production by CD4^+^ T cells is critical in regulating immune responses to avoid intestinal immune pathology in response to any potentially disease-causing microorganisms. Here, we show that during oral infection with *H. hepaticus*, Blimp-1 and c-Maf cooperate to positively regulate *Il10* gene expression and differentially negatively regulate a large network of proinflammatory effector genes. *H. hepaticus* infection of double-deficient *Prdm1*^fl/fl^*Maf*^fl/fl^*Cd4*^Cre^ mice resulted in a high-level type 1 immune response in LPLs with increased *Ifng, Csf2, Il23r*, *ll12rb1* and *Il12rb2* expression, which were also increased (but to a lesser extent) in LPLs from infected *Prdm1*^fl/fl^*Cd4*^Cre^ and *Maf*^fl/fl^*Cd4*^Cre^ mice. Conversely, *H. hepaticus* infection of *Maf*^fl/fl^*Cd4*^Cre^ mice resulted in a Type 17 response, with increased *Il17a* and *Il22* expression and a pronounced signature of neutrophils, myeloid cells and innate immunity. Thus, Blimp-1 and c-Maf cooperate to control common and distinct gene networks in T cells by specific, direct and shared actions on proinflammatory cytokines, over and above their direct stimulatory effects on *Il10*. Genes over-expressed in human IBD showed differential expression in the LPLs from *H. hepaticus*-infected mice with T cell-specific deletion of *Prdm1* or *Maf*, potentially revealing T cell-regulated mechanisms relevant to human disease.

Our findings that T cell-specific deletion of *Maf* did not result in overt inflammation of the colon in the steady state are in keeping with most reports that *Maf*^fl/fl^*Cd4*^Cre^ or *Maf*^fl/fl^*Foxp3*^Cre^ mice do not develop colitis^13,14,17,18^ in the absence of challenge, although there are a few conflicting reports of spontaneous mild colitis^18,19^. Likewise, there are conflicting reports in T cell-specific *Prdm1*-deficient mice, with some indicating spontaneous colitis^22,23,31^ whereas in other reports no signs of colitis were observed^24^, the latter in keeping with our findings. Spontaneous colitis in double-deficient *Prdm1*^fl/fl^*Maf*^fl/fl^*Cd4*^Cre^ mice has been reported^14^, in contrast to our findings in the same mice, probably reflecting the controlled microbiota in our vivarium. Spontaneous colitis in mice with T cell-specific deletion of *Prdm1, Maf* or both transcription factors has been associated with either T_reg_ cells losing their immunosuppressive function^14^ or increased frequencies of T_H_17 cells^23^, probably triggered by undefined microbiota or infection with pathobionts. Microbiota or pathobionts may be maintaining T_H_17 cells at the steady state, as has been previously described^17,32,33^ and very recently shown to be under c-Maf-dependent T_reg_ control^17^.

Given that mice with T cell-specific deletion of *Prdm1, Maf* or both transcription factors did not exhibit any signs of intestinal inflammation, this provided us with an excellent baseline to determine their role in regulating the intestinal immune response to a defined pathobiont. No intestinal pathology was observed in the control *H. hepaticus*-infected mice unless they were co-administered anti-IL-10R mAb, in keeping with previous reports^8,34–36^. As no increase in pathology was observed in the *Prdm1*^fl/fl^*Maf*^fl/fl^*Cd4*^Cre^ *H. hepaticus*-infected mice when co-administered anti-IL-10R mAb, this suggests that the high level of intestinal pathology resulted from the effects of both *Prdm1* and *Maf* on other immune factors in addition to their co-dominant and direct role in *Il10* gene regulation^3–5,13,14,37^. This is supported by our findings of distinct Blimp-1 binding sites for *Ifng* and unique c-Maf binding sites for *Il17a* and *Il22*, coinciding with open chromatin (ATAC-seq) sites from sorted CD4^+^ T cells from *H. hepaticus*-infected mice.

Our findings that T cell-associated and ILC-associated genes such as *IFNG, IL12RB2, IL23R*, *IL18R, CSF2, IL17A* and *IL17F* that were highly expressed in colon biopsies from human IBD^11,38,39^ showed differential expression in the LPLs from double-deficient *Prdm1*^fl/fl^*Maf*^fl/fl^*Cd4*^Cre^ and *Maf*^fl/fl^*Cd4*^Cre^ mice and were enriched in Foxp3^−^ effector T cells and ILC may reveal distinct pathological mechanisms of human IBD. Many of these genes have been reported as susceptibility loci for IBD and have highlighted shared genetic risk across populations^11,39^. For example, *Prdm1* has been reported to harbor rare missense mutations in PR domain-containing 1 (PRDM1) associated with IBD, and these mutations resulted in increased T cell proliferation and production of proinflammatory cytokines such as IFNγ^12^. Moreover, the Notch–STAT3^−^–Blimp-1–c-Maf axis, shown to be a common anti-inflammatory pathway in T cells, has been shown to be defective in effector CD4^+^ T cells from patients with Crohn’s disease^40^.

The increased *Ifng* and *Csf2* expression accompanying the intestinal pathology in the *Prdm1*^fl/fl^*Maf*^fl/fl^*Cd4*^Cre^ *H. hepaticus*-infected mice, and to a lesser extent in the single T cell-specific transcription-factor-deficient mice, supports earlier studies in different models of colitis, implicating IFNγ^41^ and GM-CSF^42^ in exacerbating intestinal pathology. Although IL-23 receptor signaling has been strongly linked with induction of a T_H_17 response^43^, expression of *Il23r* in LPLs from *Prdm1*^fl/fl^*Cd4*^Cre^ and *Prdm1*^fl/fl^*Maf*^fl/fl^*Cd4*^Cre^ mice accompanying high levels of *Ifng*, but not in the Type 17-dominated response in *Maf*^fl/fl^*Cd4*^Cre^ mice, supports earlier reports of IL-23 in promoting IFNγ production and intestinal inflammation^35,44^. The significant increase in *Il17a* expression in the LPLs from *H. hepaticus*-infected *Maf*^fl/fl^*Cd4*^Cre^ mice supported a major role for c-Maf in negatively regulating IL-17 responses to pathobionts, in keeping with previous in vitro reports highlighting c-Maf as a repressor of T_H_17 (*Il17a* and *Il22)* responses^16,45^. Given that CD4^+^ T cells from LPLs of *H. hepaticus*-infected control mice showed a similar level of expression of *Il17a*, although not *Il22*, to that in *Maf*^fl/fl^*Cd4*^Cre^ mice, this suggests that microbiota may be maintaining *Il17a* expression at the steady state as has been previously described^17,32,33^. The source of the increased *Il17a* and *Il22* in the total LPLs from *H. hepaticus Maf*^fl/fl^*Cd4*^Cre^ mice above that of infected control mice may be attributed to the increased abundance of T_H_17 cells or to other IL-17A producers such as ILC3 or γδ-T cells^34^ and to the heterogeneity of T_H_17 cells.

Increased *Il17a* expression in the *Maf*^fl/fl^*Cd4*^Cre^ mice may explain the increased numbers of neutrophils in the colon, as IL-17 has been reported to promote neutrophil recruitment and function during infections, and supports the role of neutrophils in intestinal pathology and their association with human IBD^29^. Other neutrophil/myeloid-associated genes, *PROK2* (prokineticin 2) and *FCGR3*, which were most highly expressed in the biopsies from ulcerative colitis patients, were most abundant in the LPLs from *H. hepaticus*-infected *Maf*^fl/fl^*Cd4*^Cre^ mice, potentially underpinning a pathobiologic neutrophil-driven mechanism for human IBD. Cytokines previously associated with IBD, such as *Il1a*^28^, *Osm*^11,46^ and other myeloid-associated genes were elevated in the LPLs from *H. hepaticus*-infected *Maf*^fl/fl^*Cd4*^Cre^ but to a lesser extent in double-deficient *Prdm1*^fl/fl^*Maf*^fl/fl^*Cd4*^Cre^ infected mice. Given that neutrophil numbers, neutrophils and innate immunity-associated genes were increased to a lesser extent in the double-deficient *Prdm1*^fl/fl^*Maf*^fl/fl^*Cd4*^Cre^ infected mice, this may suggest cross-regulation of the IL-17 responses and neutrophil function by IFNγ^47^ or by other factors elevated in the absence of *Prdm1* in T cells.

Our findings support previous reports that Foxp3^+^ T_reg_ cells are the major source of *Il10* during *H. hepaticus* infection and are most likely to control intestinal inflammation. Foxp3^+^RORγt^+^ T_reg_ cells were almost completely abolished in LPLs from infected *Maf*^fl/fl^*Cd4*^Cre^ mice, as previously reported^27^, but not in the infected double-deficient *Prdm1*^fl/fl^*Maf*^fl/^^fl^*Cd4*^Cre^ mice, which exhibited the maximum pathology and expressed the lowest levels of *Il10* in CD4^+^ T cells, indicating that Foxp3^+^RORγt^+^ T cells are not the only IL-10 producing Foxp3^+^ T_reg_ population regulating intestinal pathology. The source of low levels of Blimp-1 and c-Maf-dependent *Il10* expression by Foxp3^−^ T cells could potentially be T_H_ cells or Tr1 cells^18,48^, which could have arisen by chronic stimulation of T_H_17 or T_H_1 cells as previously reported^49^ or from peripherally induced T_reg_ cells that have lost *Foxp3* expression^50^. The CD4^+^ T cell source of IL-10 controlling immune responses to limit host damage is likely to be dictated by whether the immune response is to intestinal microbiota, pathobionts, different pathogens or different isolates of the same pathogen, and may change at different stages of infection and/or anatomical locations, as we previously discussed^4^.

Intestinal IL-10-producing T_reg_ cells, which exert their effects in lymphoid aggregates in the lamina propria, have been recently referred to as Foxp3^+^ T_reg_ effector cells, expressing *Areg, Gzmb, Icos, Tigit, Tnfrsf4* (OX40) and *Tnfrsf18* (GITR)^34^. Although our findings support the increased expression of these effector genes in Foxp3^+^ T_reg_ cells, these genes were increased to a much greater extent in Foxp3^−^ CD4^+^ T cells in LPLs from *H. hepaticus*-infected *Prdm1*^fl/fl^*Cd4*^Cre^, *Maf*^fl/fl^*Cd4*^Cre^ and double-deficient *Prdm1*^fl/fl^*Maf*^fl/fl^*Cd4*^Cre^ mice compared to controls (data not shown). Our findings are in keeping with previous reports that c-Maf controls T_reg_ cell-derived IL-10 and intestinal T_H_17 responses^17,18^. However, given that Foxp3^−^ c-Maf-deficient T cells expressed the maximal levels of *Il17* in the LPLs from *H. hepaticus*-infected *Maf*^fl/fl^*Cd4*^Cre^ mice, our data suggest that c-Maf is controlling *Il17* expression in non-T_reg_ IL-17-producing effector T cells.

Collectively, our findings show that Blimp-1 and c-Maf cooperate to positively regulate *Il10* expression and to directly control genes encompassing the type 1 effector cell responses to prevent severe *H. hepaticus*-induced colitis. However, *Maf* uniquely regulated increased *Il17a* and *Il22* expression and an accompanying pronounced signature of neutrophil activation and increased neutrophil numbers. Thus, Blimp-1 and c-Maf control common and distinct gene networks in T cells that regulate qualitatively different CD4^+^ T cell effector and innate immune responses and subsequent intestinal pathology. DEGs in the LPL of *H. hepaticus*-infected mice with T cell-specific deletion of *Prdm1* or *Maf* were elevated in colon biopsies from patients with IBD and may help delineate pathways to reveal novel pathobiologic mechanisms of human disease.

## Methods

### Mice

Mice were bred and maintained under specific-pathogen-free conditions in accordance with the Home Office UK Animals (Scientific Procedures) Act 1986. Age-matched male or female mice were used for experiments. *Maf*^fl/fl^ mice were provided by M. Sieweke and C. Birchmeier (Max Delbrück Centre for Molecular Medicine, Germany)^52^ and backcrossed to C57BL/6J for ten generations and then crossed to *Cd4*^Cre^ mice to generate *Maf*^fl/fl^*Cd4*^Cre^ mice as previously described^13^. *Prdm1*^fl/fl^ mice were purchased from the Jackson Laboratory, backcrossed to C57BL/6J for four generations and then crossed to *Cd4*^Cre^ mice to generate *Prdm1*^fl/fl^*Cd4*^Cre^ mice. *Prdm1*^fl/fl^*Maf*^fl/fl^*Cd4*^Cre^ and *Prdm1*^fl/fl^*Maf*^fl/fl^ control mice were generated in-house by crossing *Maf*^fl/fl^*Cd4*^Cre^ with *Prdm1*^fl/fl^*Cd4*^Cre^ mice. All animal experiments were carried out in compliance with UK Home Office regulations and were approved by The Francis Crick Institute Ethical Review Panel.

### *H. hepaticus* colitis model and antibody treatment

*H. hepaticus* (NCl-Frederick isolate 1A, strain 51449) was grown under anaerobic gas conditions 10% CO_2_, 10% H_2_/N_2_ (BOC) for 3 days on blood agar plates containing 7% laked horse blood (Thermo Scientific) and the *Campylobacter* selective supplement ‘Skirrow’ containing the antibiotics trimethoprim, vancomycin and polymyxin B (all from Oxoid). Bacteria were collected and then transferred and expanded, again under the anaerobic gas conditions above, for 3–4 days to an optical density of 0.6 in tryptone soya broth (Oxoid) supplemented with 10% FCS (Gibco) and the antibiotics mentioned above. For infection, mice received 1 × 10^8^ colony-forming units of *H. hepaticus* by oral gavage using a 22-gauge curved blunted needle on day 0 and day 1. Uninfected mice were housed in the same animal facility and only received antibody treatment. For experiments with anti-IL-10R, 1 mg of either anti-mouse IL-10R (CD210) (Clone 1B1.3A, Rat IgG1, kappa) blocking antibody or Rat IgG1 (Clone GL113, Rat IgG1) isotype control was administered on day 0 and day 7.

### Histopathology assessment

To assess the severity of colitis in *H. hepaticus*-infected mice, in addition to uninfected and steady-state aged mice (age 24–30 weeks), formalin-fixed paraffin-embedded cross-sections of proximal, middle and distal colon were stained with hematoxylin and eosin (H&E) and scored by two board-certified veterinary pathologists on a scale of 0–3 across four parameters to give a maximum score of 12. The four parameters included epithelial hyperplasia and/or goblet cell depletion, leucocyte infiltration into the lamina propria, area affected and markers of severe inflammation, which included crypt abscess formation, submucosal leucocyte infiltration, crypt branching, ulceration and fibrosis. Representative images of H&E-stained colon sections were then taken by the pathologists using a light microscope and a digital camera (Olympus BX43 and SC50).

Histopathology scoring throughout the manuscript is collated in the sections below.

For Extended Data Fig. 1, the baseline histopathology score for *Maf*^fl/fl^*CD4*^Cre^ was 0 for all 22 mice; for *Prdm1*^fl/fl^*CD4*^Cre^, the score was 0 for 16 mice, two mice had a score of 1 or 2 (reflecting very low-level histological changes) and one exceptional mouse had a score of 5 unaccounted for; for *Prdm1*^fl/fl^*Maf*^fl/fl^*CD4*^Cre^, 28 mice had a score of 0, nine mice had low-level histological changes (seven with a score of 2 and two with a score of 3) and one mouse showed a score of 4. The 36 uninfected aged fl/fl mice had a score of 0.

The following range and median of histopathology scores for the different mice infected with *H. hepaticus* are as follows: *Prdm1*^fl/fl^*Maf*^fl/fl^*CD4*^Cre^, total of 16 mice with scores of 6–11, median 8; *Maf*^fl/fl^*CD4*^Cre^, 14 mice scored 2–9 with a median of 6; *Prdm1*^fl/fl^*CD4*^Cre^, 12 mice scored 2–6 with a median of 3.5; fl/fl controls, 25 mice scored 0–3 with a median of 0, showing a consistent trend of increased colitis from the fl/fl control (no colitis) to *Prdm1*^fl/fl^*CD4*^Cre^ to *Maf*^fl/fl^*CD4*^Cre^ to *Maf*^fl/fl^*Prdm1*^fl/fl^*CD4*^Cre^ (severe colitis).

For Extended Data Fig. 6a,d, a large number of uninfected *Prdm1*^fl/fl^*Maf*^fl/fl^*CD4*^Cre^ mice (and fl/fl control mice) were needed to pool the numbers needed for performing RNA-seq and ATAC-seq on flow-sorted CD4^+^ T cells from the colon; in this case, 12 *Prdm1*^fl/fl^*Maf*^fl/fl^*CD4*^Cre^ mice were pooled in batches of four, for three biological replicates. The histopathology was examined again by two independent pathologists who reported mild changes in three out of the 12 mice; however, the changes were extremely mild, with none of the three individual mice exhibiting histopathology scores greater than two out of a maximal of 12, and the other nine exhibiting histopathology scores of 0 out of 12. The values in Extended Data Fig. 6a,d for uninfected *Prdm1*^fl/fl^*Maf*^fl/fl^*CD4*^Cre^ mice were averages of the pooled mice per replicate.

### Immunostaining of colon

Proximal, middle and distal colon formalin-fixed paraffin-embedded cross-sections were de-waxed and re-hydrated before being subjected to automated staining on the Leica BOND Rx Automated Research Stainer. Samples were treated with BOND Epitope Retrieval Solution 1 (Leica AR9961) for CD4 and CD68 antibodies, and BOND Epitope Retrieval Solution 2 (Leica AR9640) for the MPO antibody. To block endogenous peroxidase, samples were incubated in 3% hydrogen peroxide solution (Fisher chemical code H/1750/15) and 1% BSA blocking buffer (BSA Sigma-Aldrich A2153-100G, 1003353538 source SLBX0288). A multiplex panel included antibodies against CD4 (rabbit, Abcam ab183685, clone EPR19514; 1:750 dilution), CD68 (rabbit, Abcam ab283654, clone EPR23917-164; 1:2500 dilution) and MPO (goat, R&D Bio-Techne AF3667; 1:200 dilution). Leica Novolink Polymer (anti-rabbit, RE7161) was used as a secondary detection for primary antibodies raised in rabbit (CD4 and CD68) and horse anti-goat IgG polymer reagent (Impress HRP, Vector Laboratories 30036) for primary antibody MPO raised in goat. Samples were then incubated with Opal 690 (Akoya OP-001006) for CD4, Opal 520 (Akoya OP-001001) for CD68 and Opal 570 (Akoya OP-001003) for MPO, followed by DAPI nuclear counterstain (Thermo Scientific 62248; 1:2500 dilution). Slides were scanned using Akoya’s PhenoImager HT at ×20 and viewed in Akoya inForm Automated Image Analysis Software. Scanned slides were imported into QuPath (version 0.4.3) for image analysis. Cell segmentation was performed using the Stardist extension on the DAPI channel in QuPath. Machine learning was used to train object classifiers on representative regions of each experimental group for CD4, CD68 and MPO markers. Exported data was used to determine the number of positive cells per area (µm^2^) in the gut sections of the mice.

### Isolation of colon LPLs

LPLs were isolated from 1.0–1.5 cm pieces of the proximal, middle and distal colon from individual mice, which were cleaned to remove feces, opened lengthwise and transferred into Dulbecco’s PBS with no Ca^2+^ or Mg^2+^ ions (Gibco) containing 0.1% (v/v) bovine serum albumin Fraction V (Roche) (PBS + BSA). To remove the epithelium and intraepithelial lymphocytes, colonic tissue was incubated for 40 min at 37 °C with shaking at 220 rpm in 10 ml of RPMI (Lonza, BE12-702F) supplemented with 5% (v/v) heat-inactivated FCS and 5 mM EDTA (RPMI + EDTA). A second RPMI + EDTA wash was performed as above for 10 min, after which the tissue was left standing at room temperature (37 °C) in 10 ml RPMI (Lonza, BE12-702F) supplemented with 5% (v/v) heat-inactivated FCS and 15 mM HEPES (RPMI + HEPES) to neutralize the EDTA. Tissue was then digested at 37 °C with shaking at 220 rpm for 45 min in 10 ml of RPMI + HEPES with 120 µl of Collagenase VIII added at 50 mg ml^−1^ in PBS (Sigma). The 10 ml of digested tissue was then filtered through a 70 µM filter into a tube containing 10 ml of ice-cold RPMI + EDTA to neutralize the Collagenase VIII and the cells were centrifuged (1,300 rpm, 7 min, 4 °C). The resulting pellet was then resuspended in 4 ml of 37.5% Percoll (GE Healthcare), diluted in PBS + BSA from osmotically normalized stock and centrifuged (1,800 rpm, 5 min, 4 °C). After centrifugation, the pellet was recovered, resuspended in conditioned RPMI and used for subsequent analysis by flow cytometry and RNA and DNA extractions.

### Flow cytometry of colon LPLs

For the analysis of intracellular cytokine expression, isolated colon LPLs from individual mice were transferred to 48-well plates and restimulated with conditioned RPMI media containing 500 ng ml^−1^ Ionomycin (Calibiochem) and 50 ng ml^−1^ Phorbol 12-myristate 13-acetate (Sigma-Aldrich) for 2 h, after which 10 µg ml^−1^ Brefeldin A (Sigma-Aldrich) was added to each well and the cells were incubated for another 2 h. All incubations were conducted at 37 °C in a humidified incubator with 5% carbon dioxide. Following re-stimulation, LPLs were transferred into cold Dulbecco’s PBS with no Ca^2+^ or Mg^2+^ ions (Gibco). LPLs were first Fc-blocked for 15 min at 4 °C (24G2, Harlan) and then stained with extracellular antibodies: CD90.2 (53-2.1, PE, Invitrogen), CD4 (RM4-5, BV785, BioLegend), TCR-β (H57-597, APC-e780, Invitrogen), CD8 (53-6.7, BV605, BioLegend) and the UV LIVE/DEAD Fixable Blue dead cell stain (Invitrogen). LPLs were then fixed for 15 min at room temperature with 2% (v/v) formaldehyde (Sigma-Aldrich) and permeabilized for 30 min at 4 °C, using permeabilization buffer (eBioscience) and stained with the following cytokine antibodies for 30 min at 4 °C: IL-17A (eBio17B7, FITC, Invitrogen), IFNγ (XMG1.2, PE-Cy7, BD), IL-10 (JES5-16E3, APC, Invitrogen) and GM-CSF (MP1-22E9, BV421, BD). For transcription factor expression analysis, isolated LPLs remained unstimulated, were Fc-blocked and stained with the same extracellular antibodies, plus Ly6G (1A8, PE-Dazzle, BioLegend), CD11b (M1/70, eFluor450, Invitrogen), CD19 (1D3, BV711, BD Biosciences) and UV dead cell stain as with the restimulated LPLs, and then fixed for 30 min at 4 °C using FoxP3/transcription factor staining kit (eBiosciences). Following permeabilization for 30 min at 4 °C using permeabilization buffer (eBioscience), LPLs were then stained with the following transcription factor antibodies for 30 min at 4 °C: RORγt (Q31-378, AF647, BD) and Foxp3 (FJK-16s, FITC, Invitrogen). After staining, cells were resuspended in sort buffer (2% FBS in PBS + 2 mM EDTA) and analyzed on the Fortessa X20 (BD) flow cytometer. Acquired data was analyzed using FlowJo (version 10), with compensation performed using single-color controls from the cells and AbC total compensation beads (Invitrogen). Flow cytometry plots were concatenated for visualization purposes as follows. Each individual acquisition file was down-sampled to the lowest number of events per genotype, thus resulting in a final concatenated file with an even representation of each individual mouse per group. For intracellular cytokine staining, plots in Extended Data Fig. 4d,e,f, are composed of *n* = 5 for *Prdm1*^fl/fl^*Maf*^fl/fl^ and *n* = 4 for *Prdm1*^fl/fl^*Cd4*^Cre^, *Maf*^fl/fl^*Cd4*^Cre^ and *Prdm1*^fl/fl^*Maf*^fl/fl^*Cd4*^Cre^. The transcription factor staining plots in Extended Data Fig. 4m are composed of *n* = 5 for *Prdm1*^fl/fl^*Maf*^fl/fl^, *n* = 2 for *Prdm1*^fl/fl^*Cd4*^Cre^, *n* = 4 for *Maf*^fl/fl^*Cd4*^Cre^ and *n* = 5 for *Prdm1*^fl/fl^*Maf*^fl/fl^*Cd4*^Cre^. The extracellular marker staining plots in Fig. 5f are composed of *n* = 5 for *Prdm1*^fl/fl^*Maf*^fl/fl^, *n* = 5 for *Prdm1*^fl/fl^*Cd4*^Cre^, *n* = 5 for *Maf*^fl/fl^*Cd4*^Cre^ and *n* = 4–5 for *Prdm1*^fl/fl^*Maf*^fl/fl^*Cd4*^Cre^ in each uninfected and infected group.

### Sorting by flow cytometry of CD4^+^ T cells from colon lamina propria

Colon LPLs were isolated as described earlier from individual mice and the cells transferred into cold Dulbecco’s PBS (no Ca^2+^ or Mg^2+^ ions) (Gibco). Before being sorted, colon LPLs from individual mice within some experimental groups were equally pooled as follows to allow for the sorting of *n* = 3–6 biological replicates per experiment. In the uninfected groups for *Prdm1*^fl/fl^*Maf*^fl/fl^ and *Prdm1*^fl/fl^*Maf*^fl/fl^*Cd4*^Cre^, *n* = 12 mice were pooled to give three biological replicates. For the infected *Prdm1*^fl/fl^*Maf*^fl/fl^ group, *n* = 16 mice were pooled to give four biological replicates. For the following infected groups, individual mice were not pooled: *Prdm1*^fl/fl^*Maf*^fl/fl^*Cd4*^Cre^ (*n* = 6), *Prdm1*^fl/fl^*Cd4*^Cre^ (*n* = 4) and *Maf*^fl/fl^*Cd4*^Cre^ (*n* = 6). For FACS staining, LPLs were first Fc-blocked for 15 min at 4 °C (24G2, Harlan) and then stained with the extracellular antibodies CD90.2 (53-2.1, PE, Invitrogen), CD4 (RM4-5, BV785, BioLegend), TCR-β (H57-597, APC-eFluor 780, Invitrogen), CD8 (53-6.7, BV605, BioLegend) and the UV LIVE/DEAD Fixable Blue dead cell stain (Invitrogen). Live CD4^+^ T cells (CD4^+^TCR-β^+^CD90.2^+^CD8^−^) were then sorted to over 95% purity on the FACS Aria III or FACS Aria Fusion cell sorters (both BD). Sorted cells were then used for subsequent RNA and DNA extractions.

### RNA-seq of colon LPLs

RNA was extracted from colon LPLs of individual mice using the QIAShredder and RNeasy Mini Kit with on-column DNase digestion, according to the manufacturer’s instructions (Qiagen). RNA-seq libraries were then made with total RNA using the KAPA RNA HyperPrep with RiboErase and unique multiplexing indexes, according to the manufacturer’s instructions (Roche). All libraries were sequenced using the HiSeq 4000 system (Illumina) with paired-end read lengths of 100 bp and at least 25 million reads per sample.

### scRNA-seq of colon LPLs

Isolated colon LPLs from individual mice (as detailed above) were filtered using a 70 µm filter, and cells were suspended in PBS 0.04% BSA (UltraPure BSA, Invitrogen). For each sample, an aliquot of cells was stained with AO/PI Cell Viability Kit (Logos Biosystems) and counted with the LunaFx automatic cell counter. For all samples, cell viability before loading was >80%. As per the manufacturer’s instructions, the Master Mix was prepared as detailed in the Chromium Next GEM Single Cell 3′ Reagent Kit v.3.1 (Dual Index) manual, and 10,000 cells per sample were loaded into the 10× Chromium chips (10× Genomics). The 10× Chromium libraries were prepared and sequenced (paired-end reads) using the NovaSeq 6000 (Illumina).

### RNA-seq of sorted CD4^+^ T cells from colon lamina propria

RNA was extracted from flow-sorted CD4^+^ T cells isolated from the colon lamina propria of individual mice using the QIAShredder and RNeasy Mini Kit with on-column DNase digestion, according to the manufacturer’s instructions (Qiagen). RNA-seq libraries were then made with total RNA using the NEBNext Single Cell/Low Input RNA Library Prep Kit for Illumina and unique multiplexing indexes, according to the manufacturer’s instructions (New England Biolabs). All libraries were sequenced using the HiSeq 4000 system (Illumina) with paired-end read lengths of 100 bp and at least 25 million reads per sample.

### ATAC-seq of sorted CD4^+^ T cells from colon lamina propria

ATAC-seq samples from isolated LPLs were prepared as outlined in a previous publication^53^. For each sample, 50,000 cells were lysed in cold lysis buffer containing 10 mM Tris-HCl, pH 7.4, 10 mM NaCl and 3 mM MgCl_2_, 0.1% Nonidet P40 substitute (all Sigma-Aldrich), and the nuclei were incubated for 2 h at 37 °C with 50 μl of TDE1/TD transposase reaction mix (Illumina). Tagmented DNA was then purified using the MinElute kit (Qiagen) and amplified under standard ATAC PCR conditions: 72 °C for 5 min; 98 °C for 30 s and thermocycling at 98 °C for 10 s, 63 °C for 30 s and 72 °C for 1 min for 12 cycles. Each 50 μl PCR reaction consisted of 10 μl Tagmented DNA, 10 μl water, 25 μl NEBNext High-Fidelity 2× PCR Master Mix (NEB), 2.5 μl Nextera XT V2 i5 primer and 2.5 μl Nextera XT V2 i7 primer (Illumina). Nextera XT V2 primers (Illumina) were used to allow larger-scale multiplexing. These sequences were ordered directly from Sigma (0.2 scale, cartridge) and were diluted to 100 μM with 10 mM Tris-EDTA buffer, pH8 (Sigma) and then to 25 μM with DEPC-treated water (Ambion) for use in the reaction. Following amplification, ATAC-seq libraries were cleaned up using 90 μl of AMPure XP beads (Beckman Coulter) and two 80% ethanol washes while being placed on a magnetic plate stand before being eluted in 1 mM (0.1×) Tris-EDTA buffer, pH8 (Sigma-Aldrich) diluted with DEPC-treated water (Ambion). ATAC-seq libraries were then checked on the TapeStation/BioAnalyser (Agilent) before being sequenced on the HiSeq 4000 system (Illumina), with paired-end read lengths of 50 bp and at least 50–80 million uniquely mapped reads per sample.

### Statistical analysis

All figure legends show the number of independent biological experiments performed for each analysis and all replicates. Flow cytometry percentages and associated cell numbers were analyzed as a one-way ANOVA with Tukey’s multiple comparisons test and 95% confidence intervals for statistical analysis. All statistical analyses, apart from sequencing, were carried out with Prism8 software (GraphPad), and the following *P* values were considered statistically significant: *P ≤ 0.05; **P ≤ 0.01; ***P ≤ 0.001; ****P ≤ 0.0001. Analyses for RNA-seq and ATAC-seq data were performed with R version 3.6.1 and Bioconductor version 3.9. Analyses for scRNA-seq data were performed with R version 4.1 and Seurat version 4.1.1. Error bars and sample sizes used are described in the figure legends.

### RNA-seq data processing and analysis

For bulk tissue LPL RNA-seq, adaptors were trimmed using Skewer software version 0.2.2 (ref. ^54^) with the following parameters: '-m pe -q 26 -Q 28 -e -l 30 -L 100', specifying the relevant adaptor sequences. For sorted CD4^+^ T cell RNA-seq, adaptors were trimmed using FLEXBAR software^55^, as recommended by the manufacturer (NEB) when using the NEBNext Single Cell/Low input RNA Library Prep Kit for Illumina. FLEXBAR was run following the provider’s suggested pipeline found at https://github.com/nebiolabs/nebnext-single-cell-rna-seq. For both bulk tissue LPL RNA-seq and sorted CD4^+^ T cell RNA-seq, reads were aligned to the mm10 genome and the GENCODE reference transcriptome version M22 using STAR software version 2.7.1 (ref. ^56^), excluding multi-mapping reads by setting the parameter 'outFilterMultimapNmax' to 1. To increase read mapping to novel junctions, the parameter 'twopassMode' was set to 'Basic'. Raw gene counts were retrieved using QoRTs software version 1.1.8 (ref. ^57^). Normalized read counts were retrieved using DeSeq2 version 1.24.0 (ref. ^58^) and were rlog transformed to visualize gene quantifications.

### Differential gene expression of bulk tissue LPLs by RNA-seq

DeSeq2 (ref. ^58^) was used to obtain DEGs for each of the four *H. hepaticus*-infected groups: *Prdm1*^fl/fl^*Maf*^fl/fl^*, Prdm1*^fl/fl^*Cd4*^Cre^, *Maf*^fl/fl^*Cd4*^Cre^ and *Prdm1*^fl/fl^*Maf*^fl/fl^*Cd4*^Cre^ against the uninfected *Prdm1*^fl/fl^*Maf*^fl/fl^ control. A gene was considered to be statistically differentially expressed if the fold change was ≥1.5 and the BH-adjusted P value was <0.05, resulting in:

*Prdm1*^fl/fl^*Maf*^fl/fl^ infected vs uninfected *Prdm1*^fl/fl^*Maf*^fl/fl^: 5 DEGs

*Prdm1*^fl/fl^*Cd4*^Cre^ vs uninfected *Prdm1*^fl/fl^*Maf*^fl/fl^: 1,207 DEGs

*Maf*^fl/fl^*Cd4*^Cre^ vs uninfected *Prdm1*^fl/fl^*Maf*^fl/fl^: 1,740 DEGs

*Prdm1*^fl/fl^*Maf*^fl/fl^*Cd4*^Cre^ vs uninfected *Prdm1*^fl/fl^*Maf*^fl/fl^: 3,392 DEGs.

### Cell enrichment and biological pathway annotation

The identified DEGs (in any condition) were subjected to *k-*means clustering using *k* = 9; the expression values for the DEGs were standardized into *z*-scores and visualized in a heatmap (Fig. 1f). To provide a biological interpretation of these clusters, each cluster was subjected to 'cell-type enrichment' and 'biological pathways' annotation. The cell-type enrichment analysis used the cell-type signatures from a previous publication^51^, and a Fisher’s exact test was performed to identify cell-type signatures enriched in each of the clusters. Adjusted *P* values were obtained using the BH correction. Cell-type signatures that are statistically significantly enriched (adjusted *P* < 0.05) are shown in Fig. 1g. The R package topGO^59^ was used to obtain the enriched biological processes in each cluster (Extended Data Fig. 1e). Additionally, an ingenuity pathway analysis (IPA) 'core analysis' (Qiagen, www.qiagen.com/ingenuity) was performed to identify the IPA pathways enriched in each cluster. The *Prdm1*^fl/fl^*Maf*^fl/fl^*Cd4*^Cre^ versus uninfected *Prdm1*^fl/fl^*Maf*^fl/fl^ expression values served as input for the calculation of the *z*-scores in the bar plots depicted in Extended Data Fig. 1e, as this was the comparison that resulted in the highest amount of DEGs.

### Differential gene expression in sorted colonic CD4^+^ T cells

DeSeq2 (ref. ^58^) was used to obtain DEGs for the uninfected *Prdm1*^fl/fl^*Maf*^fl/fl^*Cd4*^Cre^ and each of the four *H. hepaticus*-infected groups: *Prdm1*^fl/fl^*Maf*^fl/fl^*, Prdm1*^fl/fl^*Cd4*^Cre^, *Maf*^fl/fl^*Cd4*^Cre^ and *Prdm1*^fl/fl^*Maf*^fl/fl^*Cd4*^Cre^ against the uninfected *Prdm1*^fl/fl^*Maf*^fl/fl^ control (Extended Data Fig. 6b). A gene was considered to be a statistically differentially expressed if the fold change was ≥1.5 and the BH-adjusted *P* value was <0.05.

### ATAC-seq data processing and analysis

Paired-end ATAC-seq reads from sorted CD4^+^ T cells were quality controlled and adaptors were trimmed using Skewer software version 0.2.2 (ref. ^54^) with the following parameters: '-m pe -q 26 -Q 30 -e -l 30 -L 50', specifying 'CTGTCTCTTATACAC' as reference adaptor sequence to remove. The reads were then aligned to the mm10 genome using BWA-MEM^60^, duplicate reads were removed with Picard^61^, and SAMtools 1.3.1 (ref. ^62^) was used to discard discordant alignments and/or low mapping qualities (mapQ < 30). To account for transposase insertion, reads were shifted +4 bp in the forward and −5 bp in the reverse strand; moreover, read-pairs that spanned >99 bp were excluded from further analyses as they would span nucleosomes^53^. MACS2 (version 2.1.1) was used to identify ATAC-seq peaks using the following parameters: 'parameters–keep-dup all–nomodel–shift -100–extsize 200; q-value < 0.01', to identify enrichment of Tn5 insertion sites^63^. DiffBind software version 2.0.2 (ref. ^64^) was used to generate raw counts underlying each ATAC-seq peak. Furthermore, batch correction was performed on raw counts using the RUVSeq R package^65^ to remove batch effects that resulted from independent experiments. BeCorrect software^66^ was used to generate batch-corrected bigwig files, using the outputs from RUVseq software. The resulting batch-corrected and normalized counts were used for visualization. The R package ggbio was used to plot the genome browser tracks^67^. To identify differentially accessible sites of interest for each genotype, differentially accessible sites were subjected to *k-*means clustering using *k* = 7; the normalized read counts for the differentially accessible sites were standardized into *z*-scores and visualized in a heatmap.

### ChIP-seq data processing and analysis

Publicly available c-Maf ChIP-seq raw fastq files were obtained from GSE40918 (ref. ^16^) and Blimp-1 ChIP-seq raw fastq files were obtained from GSE79339 (ref. ^68^). Trimmomatic (version 0.36) was used for quality control and to trim adaptor sequences using the following parameters: 'HEADCROP:2 TRAILING:25 MINLEN:26' (ref. ^69^). Trimmed reads were aligned to the mouse genome mm10 with Bowtie version 1.1.2 (ref. ^70^), with the parameters: 'y -m2–best–strata -S'. MACS2 (version 2.1.1) was used with default parameters to identify ChIP-seq peaks, and peaks with a *q*-value of <0.01 were defined as statistically significant binding sites. 'bamCoverage' from DeepTools (version 2.4.2) was used to normalize ChIP-seq data to RPKMs, and the R package ggbio was used to visualize the genome browser tracks^67^ together with the ATAC-seq data.

### IPA pathways

T_H_1 and T_H_17 pathways were constructed in and obtained from the IPA signaling pathways library. Log_2_(fold changes) from the differential expression analyses outlined above were overlaid on the T_H_1 and T_H_17 pathways, all with a fixed scale of −5 (blue) to 3.5 (red).

### scRNA-seq data processing and analysis

Fastq files were aligned to the mm10 transcriptome, and count matrices were generated, filtering for GEM cell barcodes (excluding GEMs with free-floating mRNA from lysed or dead cells) using Cell Ranger (version 6.1.2). Count matrices were imported into R and processed using the Seurat library (version 4.0) following the standard pipeline^71^. Low-quality cells were removed, with cells kept for further analysis if they met the following criteria: the mitochondrial content was within three standard deviations from the median, more than 500 genes were detected and more than 1,000 RNA molecules were detected. DoubletFinder was used to identify doublets, assuming a theoretical doublet rate of 7.5%^72^. All samples were integrated using the CCA method, implemented by Seurat’s functions FindIntegrationAnchors() and IntegrateData(), using the top 10,000 variable features and the first 50 principal components. A total of 23 clusters were identified from the integrated dataset and were annotated with the scMCA R library (version 0.2.0) using the single-cell Mouse Cell Atlas as a ref. ^73^ and the clustifyr R library using the Immgen reference dataset (version 1.5.1). A final manually curated annotation was assigned to clusters based on scMCA and clustifyr results, and this resulted in the annotation of 17 distinct cell clusters. Marker genes for these cell clusters were identified using a Wilcoxon rank-sum test, comparing each cluster to all other clusters, and statistically significant genes (adjusted *P* < 0.05 and log_2_(fold change) > 0) were kept for further analysis.

### CellChat analysis

Cell-to-cell crosstalk was inferred using the R library CellChat (version 1.1.3)^74^ and the CellChat mouse database. The CellChat analysis was performed as outlined in the CellChat software manual, with the 'population.size' parameter set to TRUE when computing the communication probability between clusters.

### Human IBD RNA expression data analysis

Publicly available human IBD RNA expression datasets were obtained from GSE193677 (adult^38^) and GSE126124 (paediatric^75^) and were downloaded and RMA-normalized using the limma package (version 3.50.0). From both datasets, only colon biopsies from healthy controls and colon biopsies from untreated patients were used for further analysis. Normalized log_2_ expression values of the top 10,000 genes based on variances per dataset were used as input to WGNA (version 1.72.1)^76^. Gene set modules were detected using a minimum module size of 30, and a deep.split of 2 for both datasets. This resulted in 12 modules for GSE193677 and 18 modules for GSE126124. Gene Ontology biological processes enriched in the modules were annotated using clusterProfiler (version 4.0.5). Using clusterProfiler results and manual curation, a final annotation for some key modules was assigned. Furthermore, modules were tested with Qusage (version 2.26.0) using normalized log_2_ expression values as input for reciprocal datasets; statistically significant modules (adjusted *P* < 0.05) were plotted using the ggcorrplot function in R. Genes within the modules derived from WGCNA were converted to mouse gene symbols using the bioMart (version 2.56.1) R library, and genes not expressed in our mouse scRNA-seq dataset generated herein were filtered out. The remaining WGNA module genes were scored in our mouse scRNA-seq dataset using the AddModuleScore() function implemented in the Seurat R library.

### Reporting summary

Further information on research design is available in the Nature Portfolio Reporting Summary linked to this article.

## Online content

Any methods, additional references, Nature Portfolio reporting summaries, source data, extended data, supplementary information, acknowledgements, peer review information; details of author contributions and competing interests; and statements of data and code availability are available at 10.1038/s41590-024-01814-z.

### Supplementary information

Reporting Summary

Supplementary Table 1 Supplementary Table 1.

Supplementary Table 2 Supplementary Table 2.

Supplementary Table 3 Supplementary Table 3.

Supplementary Table 4 Supplementary Table 4.

Supplementary Table 5 Supplementary Table 5.

Supplementary Table 6 Supplementary Table 6.

Supplementary Table 7 Supplementary Table 7.

Supplementary Table 8 Supplementary Table 8.

## Data Availability

The materials, data and any associated protocols that support the findings of this study are available from the corresponding author upon request. The RNA-seq datasets have been deposited in the NCBI GEO database under primary accession number GSE240422. Publicly available datasets used in this study include GSE40918 (c-Maf ChIP-seq), GSE79339 (Blimp-1 ChIP-seq), GSE193677 (adult IBD) and GSE126124 (pediatric IBD).
