# Supplementary material for: Blimp-1 and c-Maf regulate immune gene networks to protect against distinct pathways of pathobiont-induced colitis
Source: Nat Immunol. 2024 Apr 12;25(5):886–901. doi: 10.1038/s41590-024-01814-z (PMC11065689; doi:10.1038/s41590-024-01814-z)
Supplement: Supplementary file 1 — Reporting Summary [file 41590_2024_1814_MOESM1_ESM.pdf]

nature portfolio

Corresponding author(s): Anne O'Garra

Last updated by author(s): Nov 28, 2023

Reporting Summary

Nature Portfolio wishes to improve the reproducibility of the work that we publish. This form provides structure for consistency and transparency in reporting. For further information on Nature Portfolio policies, see our Editorial Policies and the Editorial Policy Checklist.

Statistics

For all statistical analyses, confirm that the following items are present in the figure legend, table legend, main text, or Methods section.

n/a	Confirmed	
☐	☒	The exact sample size (*n*) for each experimental group/condition, given as a discrete number and unit of measurement
☐	☒	A statement on whether measurements were taken from distinct samples or whether the same sample was measured repeatedly
☐	☒	The statistical test(s) used AND whether they are one- or two-sided<br>*Only common tests should be described solely by name; describe more complex techniques in the Methods section.*
☐	☒	A description of all covariates tested
☐	☒	A description of any assumptions or corrections, such as tests of normality and adjustment for multiple comparisons
☐	☒	A full description of the statistical parameters including central tendency (e.g. means) or other basic estimates (e.g. regression coefficient) AND variation (e.g. standard deviation) or associated estimates of uncertainty (e.g. confidence intervals)
☐	☒	For null hypothesis testing, the test statistic (e.g. *F*, *t*, *r*) with confidence intervals, effect sizes, degrees of freedom and *P* value noted<br>*Give P values as exact values whenever suitable.*
☒	☐	For Bayesian analysis, information on the choice of priors and Markov chain Monte Carlo settings
☐	☒	For hierarchical and complex designs, identification of the appropriate level for tests and full reporting of outcomes
☐	☒	Estimates of effect sizes (e.g. Cohen's *d*, Pearson's *r*), indicating how they were calculated

Our web collection on statistics for biologists contains articles on many of the points above.

Software and code

Policy information about availability of computer code

Data collection

Depending on the cytometer, flow data was collected using either Diva or BD FACSuiteTM (BD LSR II, BD LSRFortessaTM, BD Fusion or BD FACSVerse) Softwares. All data were analysed using FlowJo software (Treestar). Histology: H&E stained colon sections were then taken by the pathologists using a light microscope and a digital camera (Olympus BX43 and SC50). OlyVIA software was used to view and score histology slides. Immunofluorescence: Slides were scanned using Akoya's PhenoImager HT at 20x and viewed in Akoya inForm Automated Image Analysis Software. All statistical analyses, apart from sequencing were carried out with Prism8 software.

Data analysis

Analyses for scRNA-seq were performed with R version 4.1. and with Seurat version Seurat_4.1.1. Bulk tissue RNA-seq and sorted CD4+ RNA-seq and ATAC-seq data analyses were performed with R version 3.6.1 and Bioconductor version 3.9. See methods section for detailed use of each software

RNA-Seq data:
Skewer 0.2.2 or FLEXBAR sorftare: used for quality controlled and adapters trimming of sequencing reads
STAR 2.7.1: used to align reads to mm10 genome and the GENCODE reference transcriptome version M22.
QoRTs 1.1.8: used to obtain "raw" gene counts.
DeSeq2 1.24.0: used to normalize read counts and obtain differentially expressed genes.
DeepTools 2.4.2: "bamCoverage" command was to retrieve RPKM normalised bigwig files.
scRNA-seq data:
CellRanger 6.12: to align reads to mm10 transcriptome, to generate count matrices, and initial filtering of empty GEM with free floating mRNA.
Seurat 4.0: to further process, QC, integrate and plot scRNA-seq data.
DoubletFinder: was used to identify doublets.
scMCA 0.2.0 and clustifyr: used to annotate cell types in the scRNA-seq data.
CellChat 1.1.3: used to identify putative cell-to-cell interactions
ATAC-seq data:

Skewer 0.2.2: used for quality controlled and adapters trimming of sequencing reads.
BWA-MEM: used to map pair-end reads to mm10 genome.
SAMtools 1.3.1: used for alignment QC (discarded alignments with a mapQ<30).
Picard: used to remove duplicate reads.
BEDTools 2.26.0: used to convert alignments from bam to bed format.
Awk: used to remove reads aligning to mitochondrial DNA, to shift reads in the forward strand by +4bp or reverse strand by -5bp, and to remove fragments with a size >99bp.
MACS2 2.1.1: used for peak-calling, in order to identify open chromatin regions
Diffbind 2.0.2: used to test for differential changes in chromatin accessibility.
DeepTools 2.4.2: "bamCoverage" command was to retrieve RPKM normalised bigwig files.
ChIP-Seq data:
Trimmomatic 0.36: used for quality controlled and adapters trimming of sequencing reads (single-end reads).
Bowtie 1.1.2: used to map single-end reads to mm10 genome.
MACS2 2.1.1: used for peak-calling, in order to identify binding sites of Blimp-1 or c-Maf

For manuscripts utilizing custom algorithms or software that are central to the research but not yet described in published literature, software must be made available to editors and reviewers. We strongly encourage code deposition in a community repository (e.g. GitHub). See the Nature Portfolio guidelines for submitting code & software for further information.

Data

Policy information about availability of data

All manuscripts must include a data availability statement. This statement should provide the following information, where applicable:

- Accession codes, unique identifiers, or web links for publicly available datasets
- A description of any restrictions on data availability
- For clinical datasets or third party data, please ensure that the statement adheres to our policy

The materials, data and any associated protocols that support the findings of this study are available from the corresponding author upon request. The RNA-seq datasets have been deposited in the NCBI Gene Expression Omnibus (GEO) database with the primary accession number GSE----
Publicly available datasets used in this study include: GSE193677,GSE126124 , GSE40918, and GSE79339.

Field-specific reporting

Please select the one below that is the best fit for your research. If you are not sure, read the appropriate sections before making your selection.

☒ Life sciences ☐ Behavioural & social sciences ☐ Ecological, evolutionary & environmental sciences

For a reference copy of the document with all sections, see nature.com/documents/nr-reporting-summary-flat.pdf

Life sciences study design

All studies must disclose on these points even when the disclosure is negative.

Sample size	Animal sample size estimates were determined using previous studies and/or pilot studies using 4-5 animals per group and guided by the 3R principle.
Data exclusions	No data exclusion was performed in this study
Replication	Unless otherwise stated, all experimental biological replicates were included in analysis e.g. in RNA-Seq and ATAC-Seq analysis with appropriate statistical methods applied. Where representative data are shown, the experimental findings were reproduced with similar results.
Randomization	Randomization was not carried out in this study. Animals were age and sex matched between experimental groups within each experiment in order to account for covariates.
Blinding	Blinding was performed during bioinformatic data analysis, using using unsupervised methods to identify differences in transcriptomic and genomic profiles. Blinding was not performed during data collection. For histology scoring, pathologists remained blinded until pathology scoring was completed.

Reporting for specific materials, systems and methods

We require information from authors about some types of materials, experimental systems and methods used in many studies. Here, indicate whether each material, system or method listed is relevant to your study. If you are not sure if a list item applies to your research, read the appropriate section before selecting a response.

Materials & experimental systems

n/a	Involved in the study
☐	☒ Antibodies
☒	☐ Eukaryotic cell lines
☒	☐ Palaeontology and archaeology
☐	☒ Animals and other organisms
☒	☐ Human research participants
☒	☐ Clinical data
☒	☐ Dual use research of concern

Methods

n/a	Involved in the study
☐	☒ ChIP-seq
☐	☒ Flow cytometry
☒	☐ MRI-based neuroimaging

Antibodies

Antibodies used

Antibodies used in this study are as follows:

Flow cytometry:
Name / Clone name / Catalog no. / Lot no. / dilution factor
MHC-II / M5/114.15.2 / 14-5321-85 / 4289851 / 1:50
CD4 eFluor 450 / RM4-5 / 48-0042-82 / E08484-1634 / 1:200
CD8 FITC / 53-6.7 / 11-0081-85 / E00116-1634 / 1:100
CD62L PE-Cy7 / MEL-14 / 25-0621-82 / E07577-1633 / 1:400
CD44 PE / IM7 / 12-0441-83 / E01240-1630 / 1:400
CD25 APC / PC61.5 / 17-0251-82 / E07106-1634 / 1:100
CD90.2 PE / 53-2.1 / 12-0902-82 / 2017830 / 1:600
TCRβ APC eFluor 780 / H57-597 / 1953166 / 1:200
IL-17A FITC / eBio17B7 / 11-7177-81 / E00850-1632 / 1:300
IL-10 APC / JES5-16E3 / 17-7101-82 / E07374-1632 / 1:100
CD11b eFluor 450 / M1/70 / 48-0112-82 / 2044765 / 1:200
Foxp3 FITC / FJK-16S / 11-5773-82 / 2126755 / 1:50
CD4 BV785 / RM4-5 / 100552 / B264992 / 1:200
CD8 BV605 / 53-6.7 / 100744 / B301628 / 1:100
Ly6G PE-Dazzle / 1A8 / 127648 // 1:100

Name / Clone name / Catalog no. / Lot no. / dilution factor
IFN-γ PE-Cy7 / XMG1.2 / 557649 / 02121 / 1:800
GM-CSF BV421 / MP1-22E9 / 564747 / 01490149 / 1:100
RORγt Alexa Fluor 647 / Q31-378 / 562682 / 9044710 / 1:100
All flow cytometry antibodies were validated by the manufacturer.

Immunofluorescence staining:
CD4: Rabbit, Abcam ab183685, clone EPR19514, 1:750 dilution
CD68: Rabbit, Abcam ab283654, clone EPR23917-164, 1:2500 dilution
MPO: Goat, R&D Bio-Techne AF3667, 1:200 dilution

Leica Novolink Polymer (anti-Rabbit, RE7161) was used as a secondary detection for primary antibodies raised in rabbit (CD4 and CD68)
Horse anti-goat IgG polymer reagent (Impress HRP)(Vector Laboratories 30036) for primary antibody MPO raised in goat

Validation

All flow cytometry antibodies were validated by the manufacturer and used as per the manufacturer's instructions. All cell culture antibodies were certified for cell culture by the provider and used at concentrations optimized and standardized in the lab from previous differentiation experiments using primary mouse naive CD4+ T cells.

Animals and other organisms

Policy information about studies involving animals; ARRIVE guidelines recommended for reporting animal research

Laboratory animals

Mice were bred and maintained under specific pathogen free conditions in accordance with the Home Office UK Animals (Scientific Procedures) Act 1986. Age-matched male or female mice were used for experiments, mostly at 8-16 weeks of age. Maffl/fl mice were provided by M. Sieweke and C. Birchmeier (Max Delbrück Centre for Molecular Medicine, Germany) and backcrossed to C57BL/6J for ten generations and then crossed to Cd4Cre mice to generate Maffl/flCd4Cre mice as described in16. Prdm1fl/fl mice were purchased from the Jackson Laboratory (Stock Number 008100), and further backcrossed to C57BL/6J for four generations and then crossed to Cd4Cre mice to generate Prdm1fl/flCd4Cre mice. Prdm1fl/flMaffl/flCd4Cre and Prdm1fl/flMaffl/fl control mice were generated in-house by crossing Maffl/flCd4Cre with Prdm1fl/flCd4Cre mice.

Wild animals

Study did not involve wild animals.

Field-collected samples

Study did not involve field collected samples.

Ethics oversight

All animal experiments were carried out in accordance with UK Home Office regulations, under Project License, O'Garra P5AF488B4,

Ethics oversight | and were approved by The Francis Crick Institute Ethical Review Panel.

Note that full information on the approval of the study protocol must also be provided in the manuscript.

ChIP-seq

Data deposition

☐ Confirm that both raw and final processed data have been deposited in a public database such as GEO.

☐ Confirm that you have deposited or provided access to graph files (e.g. BED files) for the called peaks.

Data access links<br>*May remain private before publication.*	-
Files in database submission	-
Genome browser session<br>(e.g. UCSC)	-

Methodology

Replicates	We used publicly available ChIP-seq datasets, from c-Maf ChIP-seq: GSE40918 and Blimp-1:GSE79339
Sequencing depth	We used publicly available ChIP-seq datasets, from c-Maf ChIP-seq: GSE40918 and Blimp-1:GSE79339
Antibodies	We used publicly available ChIP-seq datasets, from c-Maf ChIP-seq: GSE40918 and Blimp-1:GSE79339
Peak calling parameters	default parameters in MACS2 2.1.1 software
Data quality	c-Maf: 45,727 peaks and Blimp-1: 2,612 peaks
Software	MACS2 2.1.1 software

Flow Cytometry

Plots

Confirm that:

☐ The axis labels state the marker and fluorochrome used (e.g. CD4-FITC).

☒ The axis scales are clearly visible. Include numbers along axes only for bottom left plot of group (a 'group' is an analysis of identical markers).

☒ All plots are contour plots with outliers or pseudocolor plots.

☒ A numerical value for number of cells or percentage (with statistics) is provided.

Methodology

Sample preparation

Analysis of colon lamina propria leukocytes: LPL were isolated from 1.0-1.5cm pieces of the proximal, middle and distal colon from individual mice, which were cleaned to remove feces, opened up lengthwise and harvested into Dulbecco’s PBS with no Ca2+ and Mg2+ ions (Gibco) containing 0.1% (v/v) Bovine Serum Albumin Fraction V (Roche) (PBS+BSA). To remove the epithelium, colonic tissue was incubated for 40 min at 37°C with 220rpm shake in 10ml of RPMI (Lonza, BE12-702F) supplemented with 5% (v/v) heat-inactivated FCS and 5mM EDTA (RPMI+EDTA). A second RPMI+EDTA wash was performed for 10 min, after which the tissue left standing at room temperature in 10ml RPMI (Lonza, BE12-702F) supplemented with 5% (v/v) heat-inactivated FCS and 15mM HEPES (RPMI+HEPES) to neutralize the EDTA. Tissue was then digested at 37°C with 220rpm shake for 45 min in 10ml of RPMI+HEPES with 120µL of Collagenase VIII added at 50mg/ml in PBS (Sigma). The 10ml of digested tissue was then filtered through a 70µM filter into tube containing 10ml of ice-cold RPMI+EDTA to neutralize the Collagenase VIII and the cells centrifuged (1300rpm, 7 min, 4°C). The resulting pellet was then resuspended in 4ml of 37.5% Percoll (GE healthcare), diluted in PBS+BSA from osmotically normalized stock and centrifuged (1800rpm, 5 min, 4°C). After centrifugation the pellet was recovered, resuspended in conditioned RPMI and used for subsequent analysis by flow cytometry.

For the analysis of intracellular cytokine expression, isolated colon LPLs from individual mice were transferred to 48-well plates and restimulated with conditioned RPMI media containing 500ng/ml Ionomycin (Calibiochem) and 50ng/ml Phorbol 12-myristate 13-acetate (Sigma) for 2 hours, after which 10µg/ml Brefeldin A (Sigma) was added to each well and the cells incubated for another 2 hours. All incubations were done at 37°C in a humidified incubator with 5% carbon dioxide. Following re-stimulation LPLs were harvested into cold Dulbecco's PBS with no Ca2+ or Mg2+ ions (Gibco). LPLs were first Fc-blocked for 15 min at 4°C (24G2, Harlan) and then stained with extracellular antibodies: CD90.2 (53-2.1, PE, Invitrogen), CD4 (RM4-5, BV785, Biolegend), TCR-b (H57-597, APC-e780, Invitrogen), CD8 (53-6.7, BV605, Biolegend), and the UV LIVE/DEAD™ Fixable Blue dead cell stain (Invitrogen). LPLs were then fixed for 15 min at room temperature with 2% (v/v) formaldehyde (Sigma) and permeabilised for 30 min at 4°C, using permeabilization buffer (eBioscience) and stained with the following cytokine antibodies for 30 min at 4°C: IL-17A (eBio17B7, FITC, Invitrogen), IFN-g (XMG1.2, PE-Cy7, BD), IL-10 (JES5-16E3, APC,

Invitrogen), and GM-CSF (MP1-22E9, BV421, BD). For transcription factor expression analysis, isolated LPLs remained unstimulated, were Fc-blocked and stained with the same extracellular antibodies, plus Ly6G (1A8, PE-Dazzle, Biolegend), CD11b (M1/70, eFluor450, Invitrogen), CD19 (1D3, BV711, BD Biosciences) and UV dead cell stain as for the restimulated LPLs, and fixed for 30 mins at 4°C using FOXFIX kit (eBiosciences). Following permeabilization for 30 min at 4°C, using permeabilization buffer (eBioscience) LPLs were then stained with the following transcription factor antibodies for 30 min at 4°C: RORgt (Q31-378, AF647, BD) and Foxp3 (FJK-16s, FITC, Invitrogen). After staining, cells were resuspended in sort buffer and analysed on the Fortessa X20 (BD) flow cytometer. Acquired data was analysed using FlowJo v10, with compensation performed using single colour controls from the cells and AbC™ total compensation beads (Invitrogen). Flow cytometry plots were concatenated for visualization purposes as follows, each individual acquisition file was down-sampled to the lowest number of events per genotype, thus resulting in a final concatenated file with even representation of each individual mouse per group.

Instrument	Analysis of colon lamina propria leukocytes (LPL): Fortessa X20 (BD) flow cytometer. Sorting of colon LPL: BD's FACs Aria III or FACs Aria Fusion cell sorters
Software	Data was collected using either Diva Software. All data were analysed using FlowJo software v10 (Treestar). All statistical analyses for flow cytometry were carried out with Prism8 software.
Cell population abundance	For the FACs staining LPLs were first Fc-blocked for 15 min at 4°C (24G2, Harlan) and then stained with the extracellular antibodies: CD90.2 (53-2.1, PE, Invitrogen), CD4 (RM4-5, BV785, Biolegend), TCR-b (H57-597, APC-eFluor 780, Invitrogen), CD8 (53-6.7, BV605, Biolegend), and the UV LIVE/DEAD™ Fixable Blue dead cell stain (Invitrogen). Live CD4+ T cells (CD4+ TCR-b + CD90.2+ CD8-) were then sorted to over 95% purity on the FACs Aria III or FACs Aria Fusion cell sorters (both BD). Sorted cells were then used for subsequent RNA and DNA extractions
Gating strategy	Live cells were selected using a FSC-A vs Propidium iodide gate, after which doublets were eliminated using FSC-H vs FSC-A and SSC-W vs SSC-H gating. Live CD4+ T cells were then sorted based on CD4+ TCR-b + CD90.2+ CD8- marker expression Analysis of colon lamina propria leukocytes: Gating strategy for the analysis of colon LPLs can be found in the supplementary figures.

☒ Tick this box to confirm that a figure exemplifying the gating strategy is provided in the Supplementary Information.
